# A CLE11b‐CLE16 Signaling Relay Mediates Root‐Shoot‐Root Crosstalk for Drought Adaptation in Common Bean

**DOI:** 10.1002/advs.74290

**Published:** 2026-02-08

**Authors:** Xinyang Wu, Shiyuan Tao, Zhuoyi Wang, Ting Sun, Zixin Zhang, Su Yang, Min Xu, Chenyi Huang, Siyi Wang, Xubo Ke, Chenze Lu, Kang Ning, Pei Xu

**Affiliations:** ^1^ International Joint Laboratory For Agricultural Plant Phenotypic Metrology and Equipment Innovation College of Life Sciences China Jiliang University Hangzhou China

**Keywords:** common bean, CLE peptide, drought, receptor, root‐shoot communication, signal relay

## Abstract

Under soil drought conditions, plant roots sense stress and transmit signals to the shoots, leading to coordinated whole‐plant responses to the stress. While several root‐to‐shoot signals have been identified, the existence and significance of shoot‐to‐root signals in this process remain unclear. Here, we identify a two‐step, CLE peptide‐mediated root‐shoot‐root signaling relay underpinning drought adaptation in common bean. We show that *PvCLE16*, a gene predominantly expressed in leaves under well‐watered conditions, was specifically upregulated in leaves, but not roots, under moderate drought. This spatially restricted transcriptional activation was driven by the leaf‐preferentially expressed transcription factor PvTCP10. Accumulated PvCLE16 in leaves promoted stomatal closure and also translocated to the roots, where it suppressed primary root elongation and stimulated lateral root development, adaptations that collectively enhance drought resilience. PvBAM3 was identified as the primary receptor for PvCLE16. Upstream of this module, we found that drought‐induced expression of *PvCLE16* in leaves requires PvCLE11b, a root‐derived CLE peptide that moved acropetally under drought. Together, our findings reveal a novel root‐shoot‐root signaling relay, wherein root‐derived PvCLE11b functions as the upward signal to induce PvCLE16 in leaves, which subsequently acts both locally and systemically by translocating to the roots to coordinate whole‐plant drought adaptation responses.

## Graphical abstract

A novel root‐shoot‐root signaling relay, mediated by CLE peptides, coordinates drought adaptation in common bean. Root‐derived PvCLE11b translocates acropetally to leaves, inducing PvCLE16 expression via PvTCP10. Leaf‐accumulated PvCLE16 triggers stomatal closure and translocates basipetally to modulate root architecture. Both actions depend on the receptor PvBAM3 to effectively enhance whole‐plant drought resilience.

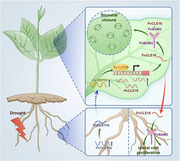

## 1 Introduction

Soil drought, a prevalent environmental stressor, poses a significant constraint on global crop production [1, 2, 3]. In response to drought stress, plants undergo a range of phenotypic adjustments to optimize water use and survival. In the aerial parts, particularly the leaves, common adaptations include stomatal closure, reduced leaf expansion, and leaf rolling, which are strategies limiting water loss through transpiration [4, 5, 6]. Below ground, root responses appear to be more nuanced and depend on the intensity of drought and the plant type. Mild to moderate drought may suppress primary root elongation while promoting lateral root proliferation to maximize access to water in the upper soil layers with minimal energy cost. Under more severe conditions, however, plants may instead prioritize deep primary root growth to tap into moisture reserves in deeper soil strata [7, 8]. While the molecular mechanisms underlying drought responses in both shoots and roots have been extensively investigated [9, 10, 11], the molecular coordination between aboveground and belowground responses remains poorly understood. It is known that, as soil moisture declines, plant roots perceive the stress and transmit signals from roots to shoots to trigger adaptive responses in aerial tissues, such as stomatal closure [12]. However, it is still unclear whether shoot‐to‐root signaling is involved in orchestrating the whole‐plant response to soil drought [13, 14].

Previous studies have often emphasized the role of Abscisic acid (ABA) as a systematic signal in plant drought responses, yet it is now increasingly viewed not as the primary long‐distance signal during soil drying, but a local inducer of stomatal closure following the reception of stress signals from roots [12]. Instead, AtCLE25 was recently identified as an earlier dehydration signal in *Arabidopsis* that moves from roots to shoots to regulate stomatal closure in an ABA‐dependent manner [15]. AtCLE25 belongs to the CLAVATA3/EMBRYO SURROUNDING REGION‐RELATED (CLE) peptides family, which comprises a diverse array of small (typically 12 to 13 amino acids in mature form), secreted signaling molecules that play pivotal roles in plant growth and development [16]. CLE peptides are initially characterized by non‐cell autonomous regulation of shoot and root meristem size, the control of flowering time, and the coordination of vascular development [17, 18, 19, 20, 21]. Later on, they are found to play a role in the modulation of responses to biotic and abiotic stresses. For instance, AtCLE9 and AtCLE10 promote the proliferation of precursors of guard cells and xylem, and mediate dehydration stress responses in guard cells to regulate stomatal closure [22, 23]. AtCLE26, which is only one amino acid different from AtCLE25, regulates lateral root formation [24] and is associated with drought stress memory [25]. It is now emerging that CLE peptides function to integrate external cues with plant development or cellular responses and therefore enable plants to adapt to a changing environment [15, 26, 27, 28, 29, 30]. CLE peptides are perceived by plasma membrane‐localized leucine‐rich repeat receptor‐like kinases (LRR‐RLKs), among which the BARELY ANY MERISTEM (BAM) family is a key component [31, 32, 33, 34]. In *Arabidopsis*, BAM1, BAM2, and BAM3 mediate diverse CLE‐dependent processes, including meristem maintenance, vascular development, and cell fate regulation [35, 36, 37]. Notably, these BAM receptors act in a tissue‐ and context‐dependent manner, allowing distinct CLE peptides to trigger specific downstream responses [33, 38].

Common bean (*Phaseolus vulgaris*) is arguably the most important grain legume for human consumption, serving as a key plant‐based protein source [39]. Predominantly grown in temperate regions, it is frequently subjected to mild to moderate drought stress. Like many other food legumes, common bean has long been considered recalcitrant to genetic transformation, which has hindered progress in elucidating the molecular mechanisms underlying key traits. Fortunately, recent advances in alternative functional genomics tools, such as highly efficient hairy root transformation and gene silencing, as well as leaf transient overexpression and silencing, have enabled reliable investigations of gene function [40, 41, 42]. Hairy root transformation produces composite plants with genetically modified roots and unaltered shoots, whereas leaf transient expression systems achieve the reverse. Together, these complementary methods serve as essential tools for studying root‐shoot interactions. In this study focused on drought adaptation mechanisms in common bean, we identified a *CLE* family gene, *PvCLE16*, as specifically upregulated in leaves under moderate soil drought. We demonstrate that in addition to locally affecting leaf phenotypes, the drought‐induced PvCLE16 also functions as a shoot‐to‐root signal to influence root architecture. We further identified another CLE peptide, PvCLE11b, as an earlier root‐derived signal that induces *PvCLE16* expression in the leaf, suggesting a root‐shoot‐root CLE peptide signaling relay. Our findings highlight a compelling example of the systemic impact of small peptide hormones enabled by their long‐distance mobility, contributing to a deeper understanding of how whole‐plant responses to soil drought stress are coordinated at the molecular level.

## 2 Results

### 2.1 *PvCLE16* is Up‐Regulated in Leaves under Moderate Soil Drought Stress

**FIGURE 1:**
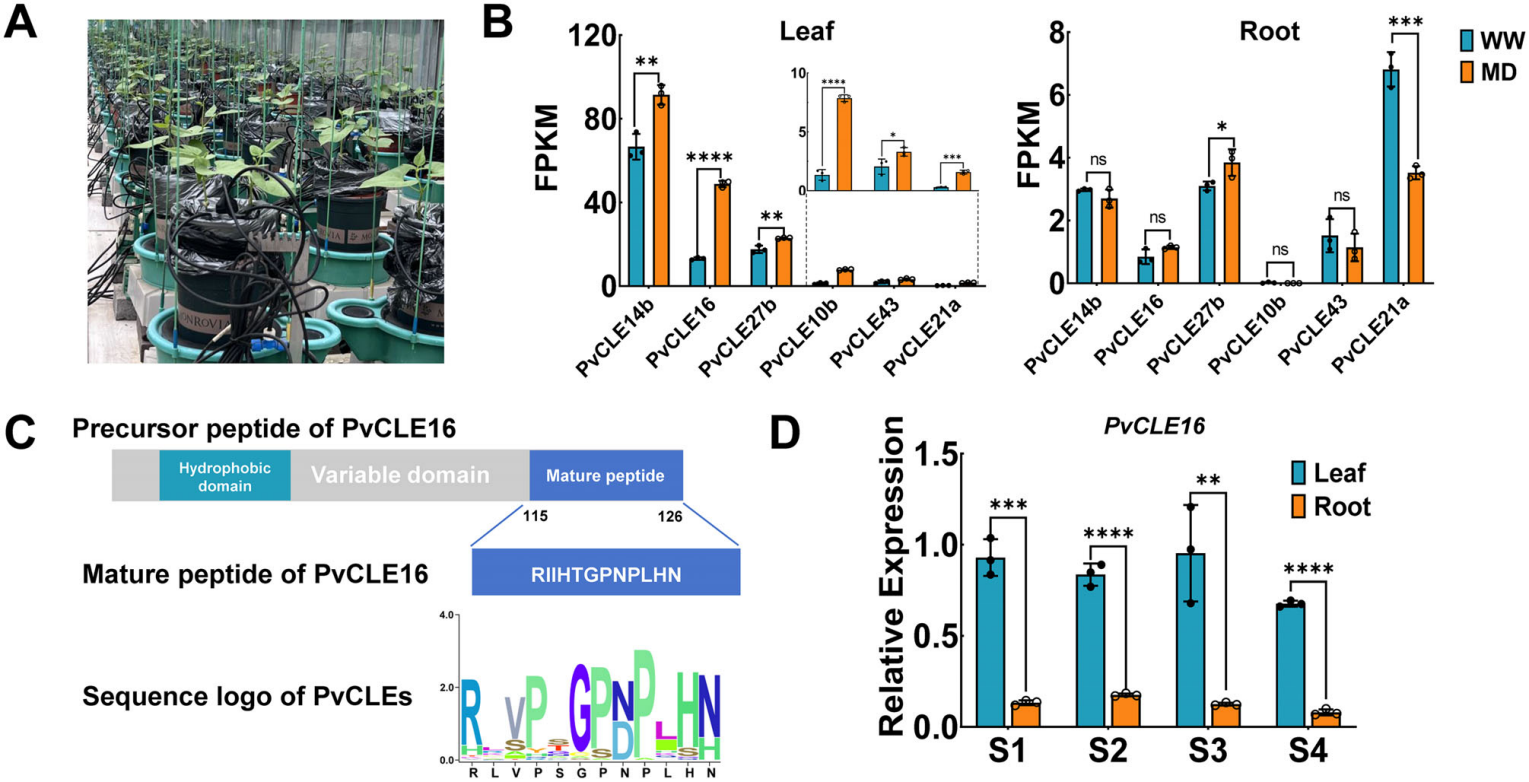
Identification of *PvCLE16* as a drought‐responsive gene. (A) The PlantArray lysimetric platform used for progressive drought experiment. Each unit integrates a gravimetric system, atmospheric and soil sensors, irrigation valves, and a controller. The system enables precise, continuous, and simultaneous measurement of the soil water content and plant transpiration rate for all units. (B) Expression profiles of *PvCLE* genes in leaves and roots of common bean with thirty‐days‐old under moderate drought conditions (VWC=0.145), as detected by RNA sequencing. Only genes differentially expressed in leaves (FPKM > 1) are shown. (C) Structural features of PvCLE16: predicted pre‐propeptide (top), mature peptide (middle), and conserved CLE motif (bottom) aligned with other CLE family members. (D) Developmental expression pattern of *PvCLE16*. Stages S1 to S4 correspond to specific developmental phases: S1‐Germination Stage; S2‐The first pair of real leaves expanded; S3‐The first pair of trifoliate leaves expanded; S4: Flowering Stage. In (B) and (D), Bar plots show means ± standard deviation (n = 3). One‐way ANOVA followed by Tukey's test (*p ≤ 0.05; **p ≤ 0.01; ***p ≤ 0.001;****p ≤ 0.0001; ns: not significant) was used for statistical analysis.

A previous study based on next‐generation genome assembly identified 44 *CLE* genes from common bean [43]. By re‐evaluating the *CLE* gene family with a more complete genome assembly (*Phaseolus vulgaris* v2.1, https://phytozome‐next.jgi.doe.gov/info/Pvulgaris_v2_1), we identified 47 *CLE* genes (Figure S1 and Table S1). To investigate their regulations under drought stress, we generated RNA‐Seq datasets from leaves and roots of common bean plants under moderate drought (MD) stage of progressive soil water depletion, with stress intensity precisely monitored using Volumetric Water Contents (VWC) measurement on the automated PlantArray lysimetric platform (Figure 1A). Six *CLE* genes including *PvCLE14b*, *PvCLE16*, *PvCLE27b*, *PvCLE10b*, *PvCLE43*, and *PvCLE21a*, exhibited significantly differential expression under MD in leaves. *PvCLE16* was particularly noteworthy, as it displays both a high expression level and a dramatic expression fold change under MD condition as compared to normal irrigation, whereas its expression in roots remained unchanged (Figure 1B, Figure S2).

*PvCLE16*, located on chromosome 2, is an intronless gene containing a 378‐base pair coding sequence (CDS) and is predicted to encode a primary peptide of 126 amino acids. The mature peptide is estimated to consist of 12 amino acids (Figure 1C). Spatiotemporal expression profiling revealed pronounced tissue‐specific expression patterns across developmental stages: consistently high transcript levels were observed in leaf tissues, whereas whole root systems maintained constitutively low expression throughout both vegetative and reproductive phases (Figure 1D).

### 2.2 PvCLE16 Acts Locally in Leaves and Systematically via Rootward Translocation to Orchestrate Drought Adaptation Responses

**FIGURE 2:**
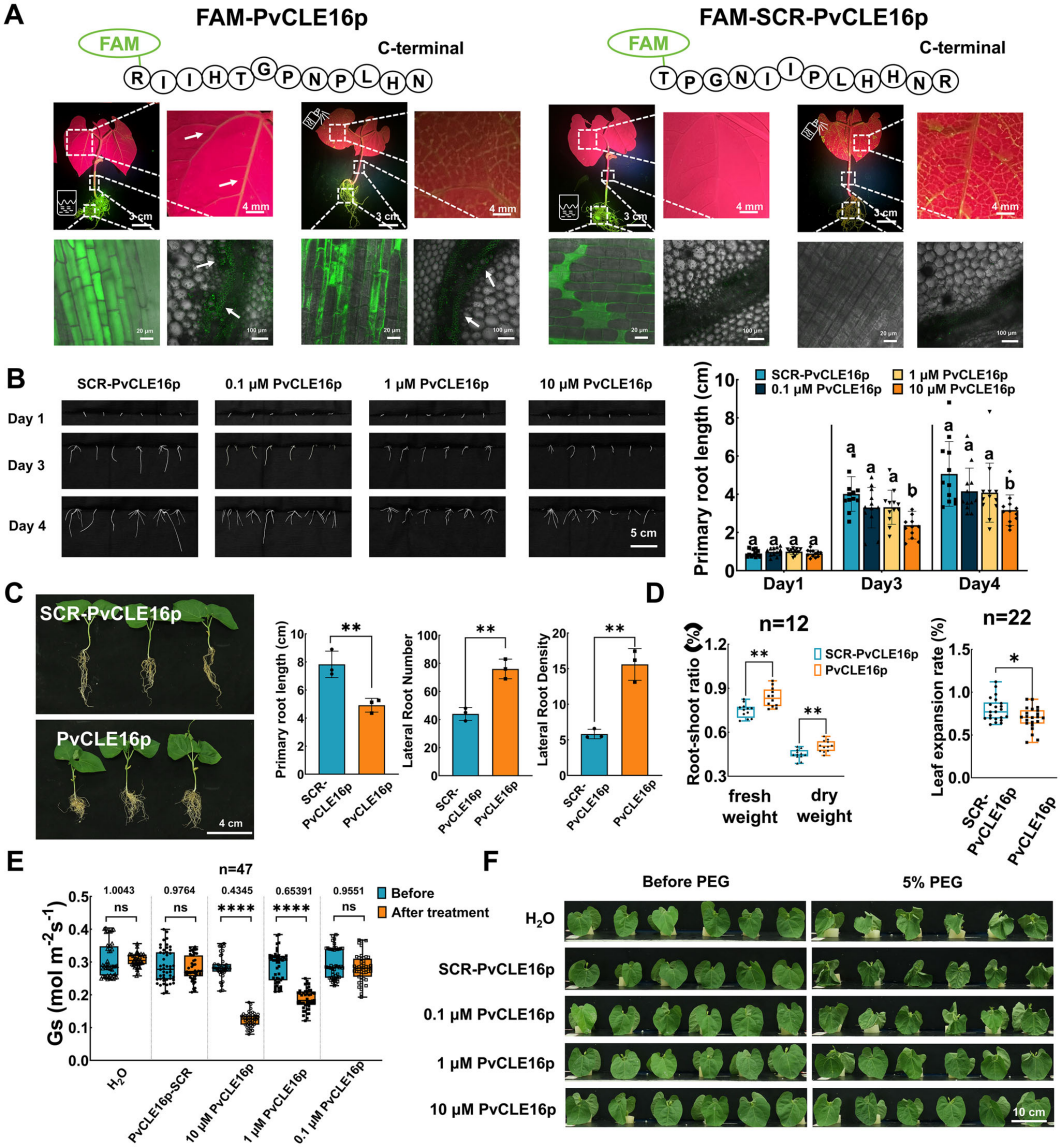
Multi‐faceted roles of PvCLE16 in regulating shoot and root responses to soil drought. Systemic fluorescence signals in common bean seedlings (root, stem and leaf) after foliar or root application of FAM‐labeled PvCLE16p (50 µM), FAM‐SCR‐labeled PvCLE16p (scrambled PvCLE16, 50 µM; negative control) for 2 h. FAM signals were visualized using a handheld fluorescent lamp or a confocal microscope. Arrows indicate vascular tissues in stems and leaf veins exhibiting FAM fluorescence signals. (B) Representative root phenotypes and quantification of primary root length in seedlings treated with 0.1, 1, and 10 µM PvCLE16p and 10 µM SCR‐PvCLE16p via root application. (C) Root morphology and quantitative analysis of primary root length, lateral root number, and lateral root density following root application of 10 µM PvCLE16p and 10 µM SCR‐PvCLE16p (negative control). (D) Root‐to‐shoot ratio and leaf expansion rate in seedlings treated with 10 µM PvCLE16p via foliar application. 10 µM SCR‐PvCLE16p was set as the negative control. (E) Stomatal aperture measurements after foliar application of 0.1, 1, and 10 µM PvCLE16p and 10 µM SCR‐PvCLE16p for 2 h. (F) Comparison of visual leaf morphology in PEG‐stressed seedlings treated in (E). In all bar charts, data represent means ± SD. In all distribution plots, the central line indicates the median, boxes represent the IQR, and whiskers indicate the data range. One‐way ANOVA followed by Tukey's test (*p ≤ 0.05; **p ≤ 0.01; ****p ≤ 0.0001; ns: not significant) was used for statistical analysis. Identical letters indicate non‐significant differences in (B).

To investigate the functions of PvCLE16, we chemically synthesized the 12‐amino‐acid mature peptide (PvCLE16p) and an N‐terminally fluorescein amidite‐labeled version (FAM‐PvCLE16p), using a non‐labeled and FAM‐labeled scrambled peptide as the negative control, respectively. FAM, as a small fluorescent probe is widely used for tracking peptide and protein movement in plant tissues. After foliar application of FAM‐PvCLE16p, fluorescence was detected by confocal microscopy within 2 h in stem vascular bundles and subsequently in roots; conversely, root application resulted in fluorescence in stem vascular tissues and leaf veins within 2 h (Figure 2A). No signal was detected with the scrambled control. These results demonstrate that PvCLE16p moves bidirectionally through vascular tissues, supporting its role as a mobile systemic signal.

**FIGURE 3:**
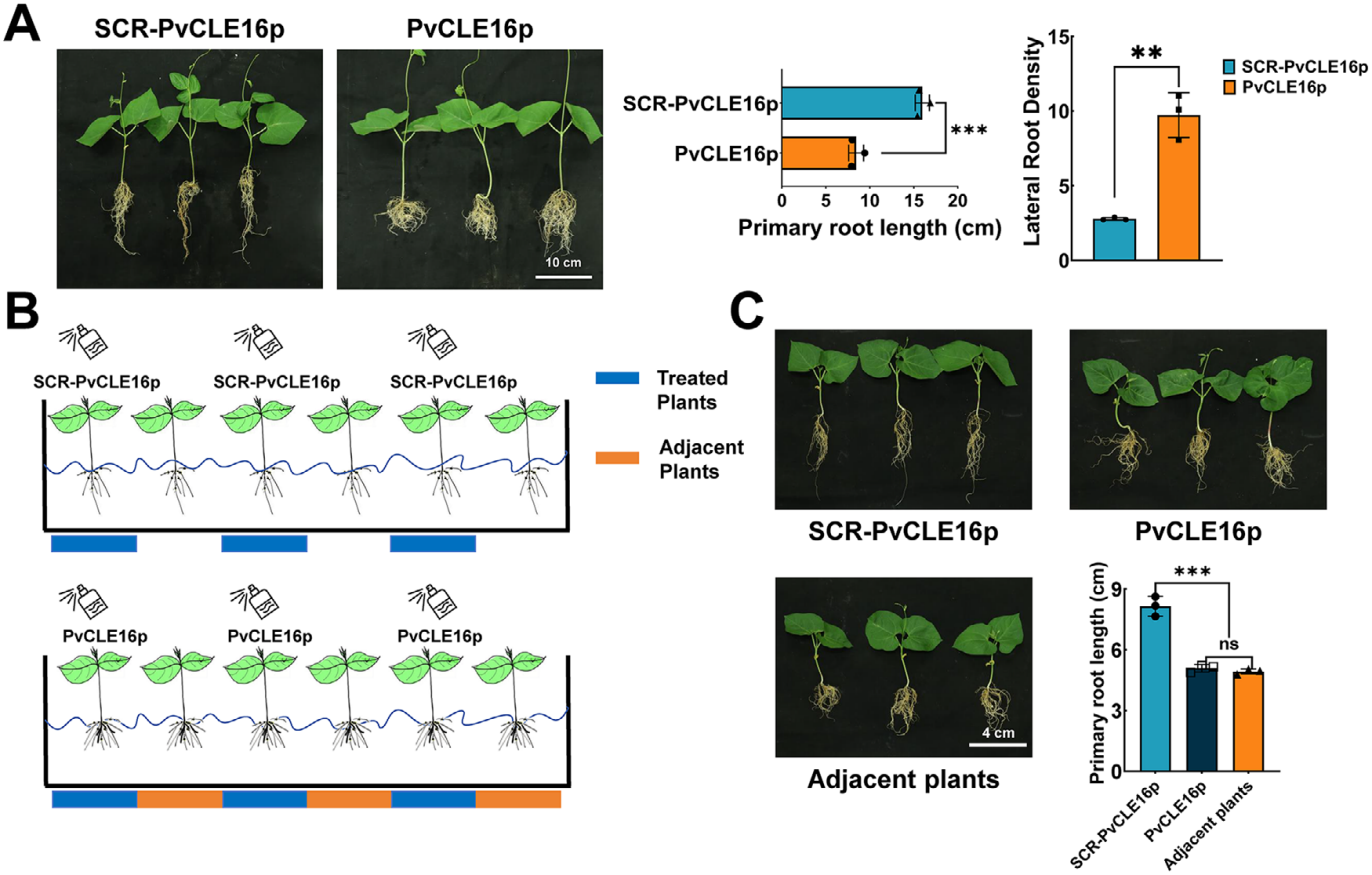
Allelopathic effects of PvCLE16p corroborating its shoot‐to‐root movability. (A) Root morphology and statistical analysis of root length after foliar application of 10 µM PvCLE16p and SCR‐PvCLE16p. (B) Schematic display of the experimental design in (C). (C) Root morphology and statistics for PvCLE16p‐treated plants and their adjacent non‐treated plants cultured in the same container, with the SCR‐PvCLE16p‐treated plants as negative control. In all panels, data represent mean ± SD. Statistical significance was determined by one‐way ANOVA with Tukey's test (**p ≤ 0.01; ***p ≤ 0.001; ns: not significant).

Functionally, root treatment with PvCLE16p significantly reduced primary root length, increased the total lateral root number, and enhanced lateral root density (lateral roots per unit primary root length), while inhibiting leaf area expansion and increasing the root‐to‐shoot biomass ratio (Figure 2B–D). These effects were dose dependent: 10 µM PvCLE16p markedly inhibited primary root growth (61.9% inhibition) and increased lateral root density, whereas 1 and 0.1 µM had no detectable effects; the scrambled peptide was inactive. In leaves, PvCLE16p induced rapid stomatal closure within 2 h (Figure 2E), and foliar application under drought stress alleviated PEG‐induced osmotic stress symptoms (Figure 2F). These leaf responses were also dose dependent, with significant effects at 1 and 10 µM but not at 0.1 µM, and no response to the scrambled control. FAM labeling did not alter PvCLE16p activity, as indicated by comparable effects on stomatal conductance (Figure S3). Furthermore, foliar application of PvCLE16p not only affected the leaf traits but had impacts on roots (Figure 3A). This effect was proved to be also allelopathic: when seedlings were hydroponically cultured in the same growth pouch, reduced root growth was observed in both the seedlings sprayed with PvCLE16p on their leaves and their neighboring untreated seedlings (Figure 3B,C), indicating that leaf‐derived PvCLE16 can be transported to roots and secreted into the surrounding medium to exert regulatory effects.

**FIGURE 4:**
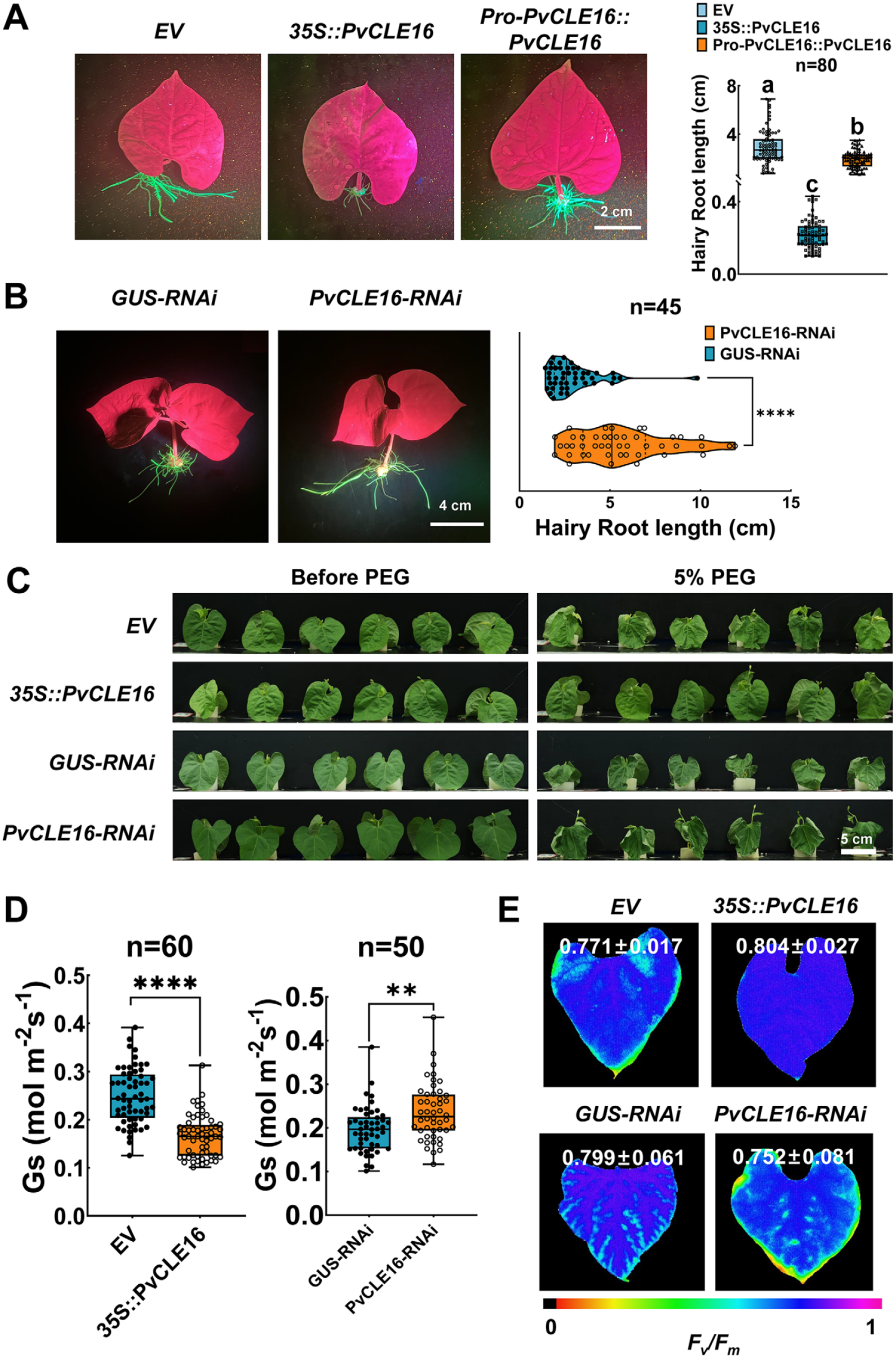
Transgenic analyses demonstrating the effects of *PvCLE16* on hairy roots and leaves. (A) Phenotypic analysis of transgenic hairy roots expressing *35S::PvCLE16*, *ProPvCLE16::PvCLE16*, and empty vector (EV, as a negative control). The vector PMDC83 contains an independent expression box to express GFP under the control of CaMV 35S promoter. Left: Representative images of hairy roots. Right: Quantification of hairy root length. (B) Phenotypic analysis of transgenic hairy roots expressing *PvCLE16‐RNAi*, and *GUS‐RNAi* (as a negative control) constructs. Left: Representative images of hairy roots. Right: Quantification of hairy root length. (C to E) Comparison of visual leaf morphology (C), Gs (D), and *F_v_/F_m_* (E)in seedlings transiently expressing *35S::PvCLE16* or *PvCLE16‐RNAi* constructs. Expression of *EV* and *GUS‐RNAi* served as the negative control. Osmotic stress was imposed by application of 5% PEG (B and D). In all distribution plots, the central line indicates the median, boxes represent the IQR, and whiskers indicate the data range. Statistical significance was determined by one‐way ANOVA followed by Tukey's test (**p ≤ 0.01; ****p ≤ 0.0001). Different letters denote statistically significant differences in (A).

To further verify PvCLE16's function, we employed transgenic approaches. We constructed a recombinant binary vector in which a separate CaMV 35S promoter independently drives the expression of *PvCLE16* and *GFP*, respectively. Overexpression of *PvCLE16* strongly inhibited emergence and elongation of transgenic hairy roots (Figure 4A). Expression driven by the native promoter (2,050 bp upstream of the CDS) produced a milder but significant inhibition, resembling the effect of PvCLE16p application (Figures 2B,C and 4A). In contrast, *PvCLE16* knockdown increased hairy root length to 192% of the control (Figure 4B). RNA‐seq analysis of *PvCLE16*‐overexpressing hairy roots identified 1,977 differentially‐expressed genes (DEGs) compared to the WT (Figure S4A). KEGG enrichment analysis revealed that these DEGs were most significantly associated with phenylpropanoid biosynthesis and plant hormone signaling, particularly auxin and jasmonic acid signaling pathways (Figure S4A). These pathways are known regulators of root growth and development [44, 45, 46]. Notably, RNA‐seq of roots following foliar PvCLE16p application revealed similar pathway enrichment, indicating that leaf‐derived PvCLE16 induces transcriptional reprogramming in roots (Figure S4A,B). Transient overexpression of *PvCLE16* in leaves induced stomatal closure, whereas RNAi‐mediated silencing increased stomatal aperture (Figure 4C,D, Figure S5A). Under 5% PEG‐6000 treatment, *PvCLE16*‐overexpressing seedlings displayed alleviated drought stress phenotypes comparable to PvCLE16p‐treated plants, while *PvCLE16* silencing exacerbated stress symptoms (Figure 4C,E, Figure S5B). Collectively, these results demonstrate that PvCLE16 functions as a mobile signal that coordinately regulates root architecture and stomatal behavior to enhance drought tolerance.

### 2.3 Leaf‐Preferentially Expressed *PvTCP10* Is Responsible for the Transcriptional Activation of *PvCLE16*

**FIGURE 5:**
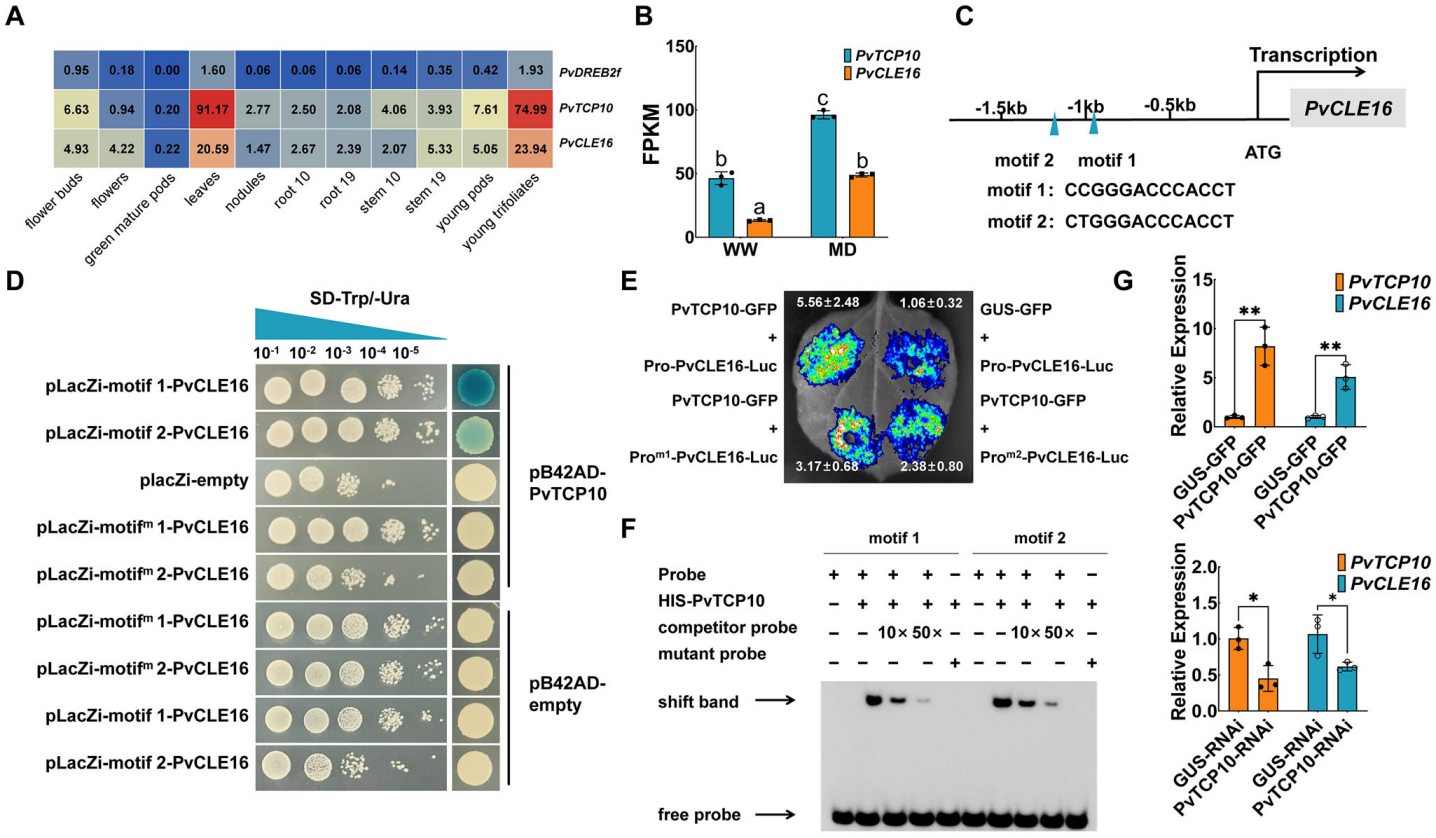
PvTCP10 directly binds to the promoter region of *PvCLE16* to regulate its transcription. (A and B) Predominant co‐expression of *PvCLE16* and *PvTCP10* in different tissues (A) and their co‐regulation by moderate drought (B). Transcript data in (A) were obtained from Phytozome (https://phytozome‐next.jgi.doe.gov/info/Pvulgaris_v2_1). (C) Predicted PvTCP10‐binding motifs in the promoter region of *PvCLE16*. Numbers indicate positions relative to the transcription start site (+1). (D) Yeast one‐hybrid assay indicating that PvTCP10 binds to both motifs with different affinities. Negative controls: empty pLacZi + pB42AD‐PvTCP10, pLacZi‐Pro1‐PvCLE16 + empty pB42AD, and pLacZi‐Pro2‐PvCLE16 + empty pB42AD. Motif^m1^:AATTTCAAACAAG, Motif^m2^:AGTTTCAAACAAG. (E) Dual‐luciferase reporter assay. Motif1 and Motif2 of *Pro‐PvCLE16* were mutated, respectively. A representative luminescence image of the *N. benthamiana* leaves 60 h after infiltration is shown. The fluorescence intensity was calculated from three independent leaves. (F) EMSA analysis using a biotin‐labeled *PvCLE16* promoter fragment containing the predicted PvTCP10‐binding motifs as the probe. Non‐labeled competitor probes with different dilutions were employed to examine binding specificity. Arrows indicate the protein–DNA complex or free probe. (G) RT‐qPCR analysis of *PvTCP10* and *PvCLE16* expression levels in leaves transiently overexpressing *PvTCP10‐GFP* (Top) and expressing *PvTCP10*‐*RNAi* constructs (Bottom). Plants expressing *GUS‐GFP* or *GUS‐RNAi* served as controls. Transcript levels were normalized to *PvUBI* (*Phvul.007G052600*). All data represent mean ± SD. Statistical significance determined by one‐way ANOVA with Tukey's test (*p < 0.05; **p < 0.01). Different letters denote statistically significant differences in (B).

To elucidate the mechanism underlying the upregulation of *PvCLE16* under drought stress, we identified the transcription factor (TF) responsible for regulating its expression. We analyzed the 2000‐bp promoter region upstream of its coding sequence using PlantRegMap for binding site identification [47], which predicted 197 potential TFs for *PvCLE16* (Table S2). Among them, only *PvTCP10* (*Phvul.011G156900*) and *PvDREB2f* (*Phvul.007G255100*) exhibited a correlated tissue expression pattern with *PvCLE16*, as demonstrated by the Phytozome co‐expression dataset (*Phaseolus vulgaris* v2.1, Figure 5A). Co‐expression relationships, whether negative or positive, are critical indicators for TF‐target gene interactions. However, *PvDREB2f* expression was consistently low across all tissues (FPKM < 2), and consequently, we focused our investigation on *PvTCP10*. *PvTCP10* exhibited a leaf‐preferential expression across tissue and an upregulation in leaves under drought similar to *PvCLE16* (Figure 5A,B), and two motifs (1: −998 to −986 bp, 2: −1146 to −1134 bp) within the *PvCLE16* promoter region were predicted as potential PvTCP10 binding sites (Figure 5C). A yeast one‐hybrid assay (Y1H) confirmed the interaction between PvTCP10 and these motifs, particularly motif 1 (Figure 5D). A dual‐luciferase reporter assay (dual‐LUC) validated that PvTCP10 enhances the transcriptional activity of the *PvCLE16* promoter (Figure 5E). This was corroborated by an electrophoretic mobility shift assay (EMSA), which demonstrated the direct binding of PvTCP10 to these motifs in the *PvCLE16* promoter (Figure 5F). Subsequent functional validation demonstrated that transient *PvTCP10‐GFP* overexpression upregulated *PvCLE16* transcript level in leaves, while RNAi‐mediated suppression of endogenous *PvTCP10* reduced its expression (Figure 5G). In addition, transient overexpression of *PvTCP10* in leaves produced phenotypes similar to *PvCLE16‐OE* seedlings under osmotic stress, including reduced stomatal conductance (Gs) and attenuated wilting symptoms compared to controls (Figure S6A). Conversely, RNAi‐mediated suppression of *PvTCP10* resulted in phenotypic changes resembling those of *PvCLE16* suppression, further supporting its role as a positive regulator of osmotic stress response and its function as a transcription factor for *PvCLE16* (Figure S6B). Together, these findings establish that the leaf‐specific *PvTCP10* is responsible for the transcriptional regulation of *PvCLE16*.

### 2.4 PvBAM3 Functions as the Primary Receptor of PvCLE16 for Drought Response

**FIGURE 6:**
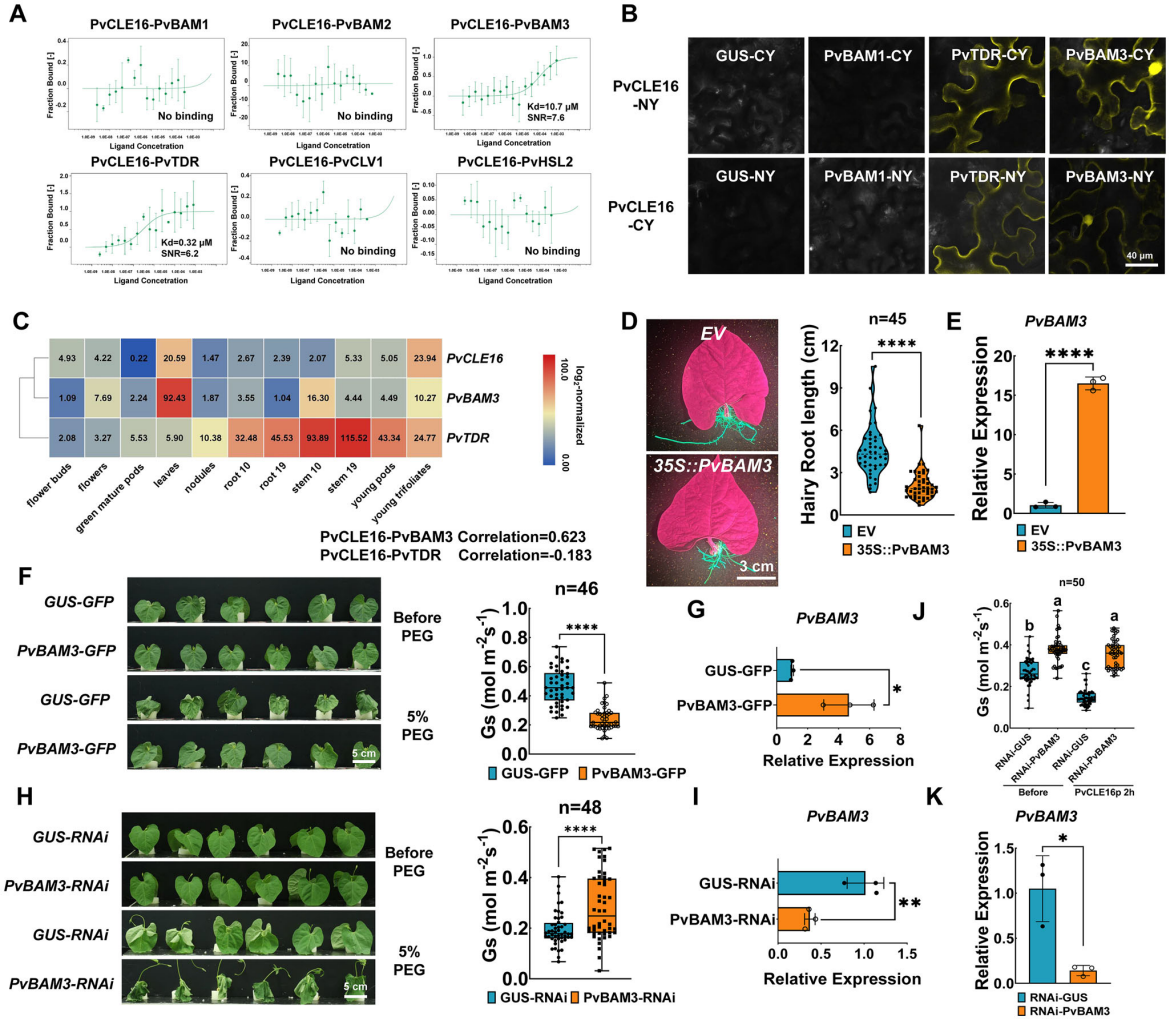
PvBAM3 functions as a primary receptor of PvCLE16. (A) MST was used to detect PvCLE16p (80 µM) binding affinity to PvBAM1‐GFP, PvBAM2‐GFP, PvBAM3‐GFP, PvTDR‐GFP, PvCLV1‐GFP and PvHSL2‐GFP. Each binding assay was repeated three times independently, and bars represent SD. (B) BiFC assays indicate an interaction between PvTDR and PvBAM3 with PvCLE16. *PvCLE16‐CY* was co‐expressed with *PvBAM3‐NY and PvTDR‐NY*, respectively, and *PvCLE16‐NY* was co‐expressed with *PvBAM3‐CY* and *PvTDR‐CY* respectively in *N. benthamiana* leaves, leading to detectable YFP fluorescence. Co‐expression with another *BAM* family member, PvBAM1 and GUS served as negative controls. (C) Co‐expression of *PvCLE16*, *PvBAM3* and *PvTDR* across different tissues. Transcript data were obtained from Phytozome (https://phytozome‐next.jgi.doe.gov/info/Pvulgaris_v2_1). (D) Morphology and the statistic of transgenic hairy roots length in hairy roots expressing 35S::*PvBAM3*, with expressing *EV* as negative control. (E) Relative expression of *PvBAM3* in common bean hairy roots in (D). (F) Comparison of visual leaf morphology and Gs in seedlings transiently expressing *PvBAM3‐GFP* constructs. Expression of *GUS‐GFP* served as the negative control. Osmotic stress was imposed by application of 5% PEG. (G) Relative expression of *PvBAM3* in common bean leaves in (F). (H) Comparison of visual leaf morphology and Gs in seedlings transiently expressing *PvBAM3‐RNAi* and *GUS‐RNAi* constructs. Osmotic stress was imposed by application of 5% PEG. (I) Relative expression of *PvBAM3* in common bean leaves in (H). (J) Gs in seedlings transiently expressing *PvBAM3‐RNAi* and *GUS‐RNAi* constructs before and after foliar application of PvCLE16 peptide for 2 h. (K) Relative expression of *PvBAM3* in common bean leaves in (J). In all bar charts, data represent means ± SD. In all distribution plots, the central line indicates the median, boxes represent the IQR, and whiskers indicate the data range. Statistical significance was determined by one‐way ANOVA followed by Tukey's test (*p ≤ 0.05; **p ≤ 0.01; ****p ≤ 0.0001). Different letters denote statistically significant differences in (J).

As a signaling molecule, PvCLE16 requires receptor binding to exert its biological functions. To identify the receptor of PvCLE16, we first conducted a genome‐wide search for common bean homologs of known *Arabidopsis* CLE receptors, given the conservation of CLE receptors across plant species. Fifteen putative PvCLE receptors were retrieved (Table S3). We next combined structural prediction, expression analysis, biochemical interaction assays, and functional validation to narrow down the candidate list. AlphaFold3‐based prediction identified six candidate receptors (PvBAM1, PvBAM2, PvBAM3, PvTDR, PvCLV1, and PvHSL2) that pass the empirical interaction confidence value (iPTM > 0.6), which were selected for further experimental validation (Figure S7). Microscale thermophoresis (MST) assays using PvCLE16p and recombinant GFP‐tagged receptor ectodomains revealed that, among the six candidates tested, only PvBAM3 and PvTDR exhibited reproducible binding to PvCLE16 (Figure 6A), with moderate‐to‐strong binding affinities within the range reported for functional CLE‐receptor pairs in plants [48, 49]. Bimolecular fluorescence complementation (BiFC) assay using GUS and PvBAM1 as negative controls confirmed the interaction between PvCLE16 and PvBAM3 and PvTDR in *Nicotiana benthamiana* cells (Figure 6B).

CLE peptides and their cognate receptor genes are often co‐expressed [50, 51]. Expression analysis revealed that *PvBAM3* exhibited significant tissue‐expression correlation with *PvCLE16* (Correlation = 0.623, *P* = 0.041), with both genes being highly expressed in leaves, whereas *PvTDR*, predominantly expressed in stems, showed no such expression correlation (Correlation = ‐0.183, *P* = 0.590) with *PvCLE16* (Figure 6C). This expression pattern of *PvTDR* is consistent with previous studies implicating a primary role for TDR in vascular development and less likely to be the receptor of *PvCLE16* in leaf responses to drought [52, 53]. To provide more functional insights, we further performed hairy root transformation experiment that showed *PvBAM3*, but not *PvTDR*, overexpression inhibited primary root growth, mirroring the phenotype observed in *PvCLE16‐OE* roots (Figure 6D,E, Figure S8). Transient overexpression of *PvBAM3 but not PvTDR* in leaves significantly alleviated osmotic‐induced wilt phenotype and reduced Gs, phenocopied the effect of *PvCLE16‐OE* (Figure 6F,G), whereas its expression knockdown (*PvBAM3‐RNAi*) led to an opposite phenotype (Figure 6H,I). Furthermore, exogenous PvCLE16p failed to induce stomatal closure or alleviate drought‐related phenotypes in *PvBAM3‐RNAi* plants (Figure 6J,K). Similarly, PvCLE16p treatment of *PvBAM3‐RNAi* hairy roots did not reproduce the typical PvCLE16p‐induced root architectural responses observed in control plants (Figure S9). Together, these findings suggest that PvBAM3 functions as the primary functional receptor mediating PvCLE16‐dependent drought responses.

### 2.5 PvCLE16‐Mediated Drought Adaptation Requires the Earlier Root‐To‐Shoot Signaling Molecule PvCLE11b

**FIGURE 7:**
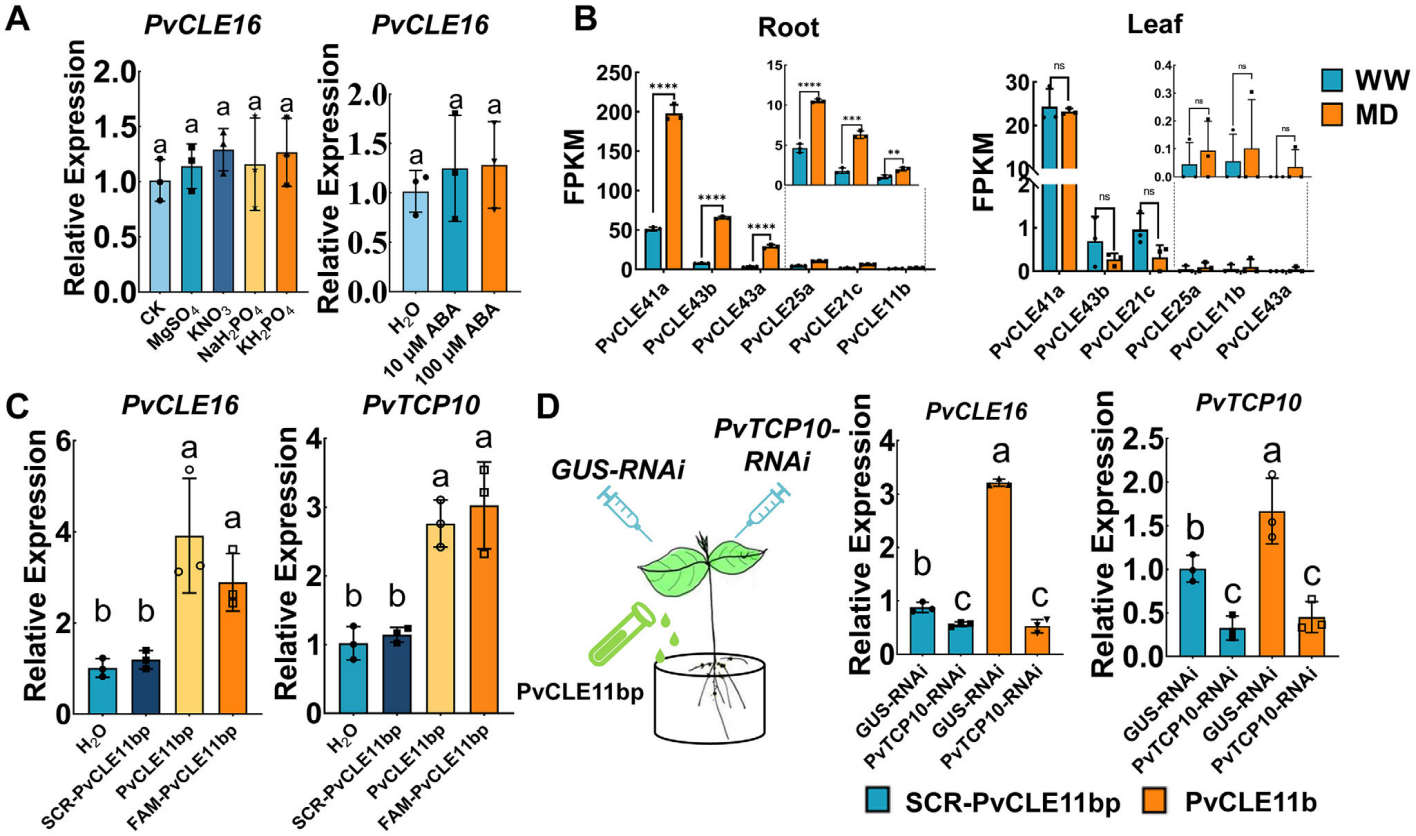
Identification of PvCLE11b as a potential root‐derived signal under soil drought stress that induces *PvCLE16* expression in leaves in a PvTCP10‐dependent manner. (A) Effects of root treatment of K^+^, SO_4_^2^^−^, PO_4_^3^^−^, NO_3_^−^, ABA on the leaf expression of *PvCLE16*. (B) Expression profiles of *PvCLE* genes in roots and leaves under drought conditions, as detected by RNA‐Seq. Only genes differentially expressed in roots are shown. (C) Effects of root application of PvCLE11bp (10 µM) on leaf expression of *PvTCP10* and *PvCLE16*. Both unlabeled and FAM‐labeled PvCLE11bp induced comparable upregulation of transcript levels in leaves. (D) Interference of *PvTCP10* expression significantly attenuated the induction of *PvCLE16* expression in leaves following root application of the PvCLE11bp (10 µM). All data represent mean ± SD. Statistical significance determined by one‐way ANOVA with Tukey's test (**p < 0.01; ***p < 0.001; ****p < 0.0001; ns, not significant). Identical letters indicate non‐significant differences in (A, C, and D).

Given that root serves as the primary organ for perceiving soil drought stress, while *PvCLE16* transcriptional activation, by PvTCP10, occurs specifically in leaves, we hypothesized that drought triggers a root‐derived signaling molecule that translocates to aerial tissues to activate the two genes’ expressions. Various signaling molecules, including K^+^, SO_4_^2^^−^, PO_4_^3^^−^, NO_3_^−^, and ABA have been proposed as candidates for mediating long‐distance acropetal signaling under drought stress [54, 55, 56, 57, 58, 59, 60, 61]. To test their involvement, we treated the roots of common bean plants with each of these chemicals and assessed *PvCLE16* expression in the leaves. However, none of these treatments had a significant effect (Figure 7A). A recent study in *Arabidopsis* have identified AtCLE25 as a drought‐triggered root‐to‐shoot signal inducing stomatal closure [15]. Inspired by this, we investigated whether a CLE peptide in common bean could similarly function as an acropetal drought signal. By analyzing the expression profiles of all *PvCLE* genes, we found six genes were upregulated in roots but not in leaves under MD, rendering them potential candidates for drought‐induced root‐to‐shoot peptide signals (Figure 7B). We synthesized the mature peptides of these six PvCLEs and examined their impact on *PvTCP10* and *PvCLE16* expression by applying each peptide to the roots (Figure 7C, Figure S10A,B). When *PvTCP10* expression was knocked down in leaves, root application of PvCLE11bp could no longer induce *PvCLE16* expression (Figure 7D). In roots, where *PvTCP10* is barely expressed, *PvCLE16* expression was also not significantly inducible by PvCLE11bp application (Figure S11). These findings indicate that root‐derived PvCLE11b activates *PvCLE16* expression in leaves in a PvTCP10‐dependent manner.

**FIGURE 8:**
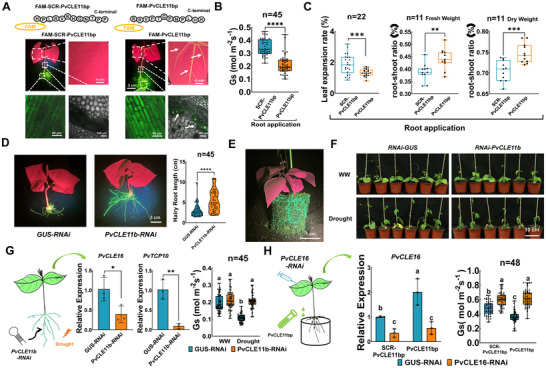
PvCLE11b acts as an earlier shootward signal initiating PvCLE16‐mediated drought adaptation. (A) Systemic fluorescence signals in common bean seedlings (root, stem, and leaf) after root application of FAM‐labeled PvCLE11bp (50 µM) or FAM‐SCR‐labeled PvCLE11bp (scrambled PvCLE11b, 50 µM; negative control) for 2 h. FAM signals were visualized using a handheld fluorescent lamp or confocal microscope. Arrows indicate vascular tissues in stems and leaf veins exhibiting FAM fluorescence signals. (B) Gs following root application of 10 µM PvCLE11bp for 2 h; 10 µM SCR‐PvCLE11bp treatment serves as the negative control. (C) Leaf expansion rate and root‐to‐shoot ration of common bean seedlings treated with 10 µM PvCLE11bp via root application. (D) Phenotypic analysis of transgenic hairy roots expressing *PvCLE11b‐RNAi* and *GUS‐RNAi* (as a negative control) constructs. Left: Representative images of hairy roots. Right: Quantification of hairy root length. (E) Representative image of the composite plants thar were transplanted from (D) to soil cultivation condition for 12 days. (F‐G) Visual phenotype (F), *PvCLE16* and *PvTCP10* expression in leaves and Gs (G) of plants in (E) following 4 days of water withholding. (H) *PvCLE16* expression and Gs in the leaves of common bean seedlings with *PvCLE16* knockdown in the leaves and PvCLE11bp (10 µM) applied to the roots. In all bar charts, data represent means ± SD. In all distribution plots, the central line indicates the median, boxes represent the IQR, and whiskers indicate the data range. ANOVA with Tukey's test (*p < 0.05; **p < 0.01; ***p < 0.001; ****p < 0.0001). Different letters denote statistically significant differences in (G and H).

To further validate the PvCLE11b‐PvCLE16 signaling relay, we confirmed the rapid acropetal transport of root‐applied PvCLE11bp to shoots using FAM fluorescence labeling (Figure 8A). The root treatment of PvCLE11bp reduced Gs, inhibited leaf area expansion, and increased the root‐to‐shoot biomass ratio, resembling the phenotype observed with PvCLE16p application to leaves (Figure 8B,C). In roots, knockdown of *PvCLE11b* led to a 181% increase in root length, a phenotype also observed when *PvCLE16* or *PvBAM3* was silenced (Figure 8D). To compare drought responses, we transplanted composite plants bearing *PvCLE11b*‐silenced hairy roots into soil for continued growth, using composite seedlings bearing *GUS‐RNAi* constructs in hairy roots as controls (Figure 8E). Functionally, this experiment is equivalent to a grafting assay. After twelve days of growth under well‐watered conditions followed by four days of water withholding, these seedlings exhibited a more severe wilting phenotype and impaired stomatal closure compared to controls (Figure 8F,G). RT‐qPCR analysis revealed that the expression of *PvCLE16* and *PvTCP10* in leaves was significantly lower in the *PvCLE11b*‐silenced composite seedlings following drought treatment (Figure 8G). These results suggest that PvCLE11b production in roots during drought is essential for initiating appropriate shoot responses. Conversely, when *PvCLE16* expression was silenced in shoots, application of PvCLE11bp to roots failed to induce stomatal closure, confirming that PvCLE11b signals through PvCLE16 rather than acting directly to trigger shoot responses (Figure 8H). Collectively, these results reveal a novel root‐shoot‐root signaling relay, wherein PvCLE11b functions as the upward signal to induce *PvCLE16* expression in leaves, which subsequently acts both locally and systemically to coordinate the whole‐plant drought adaptation responses.

## 3 Discussion

**FIGURE 9:**
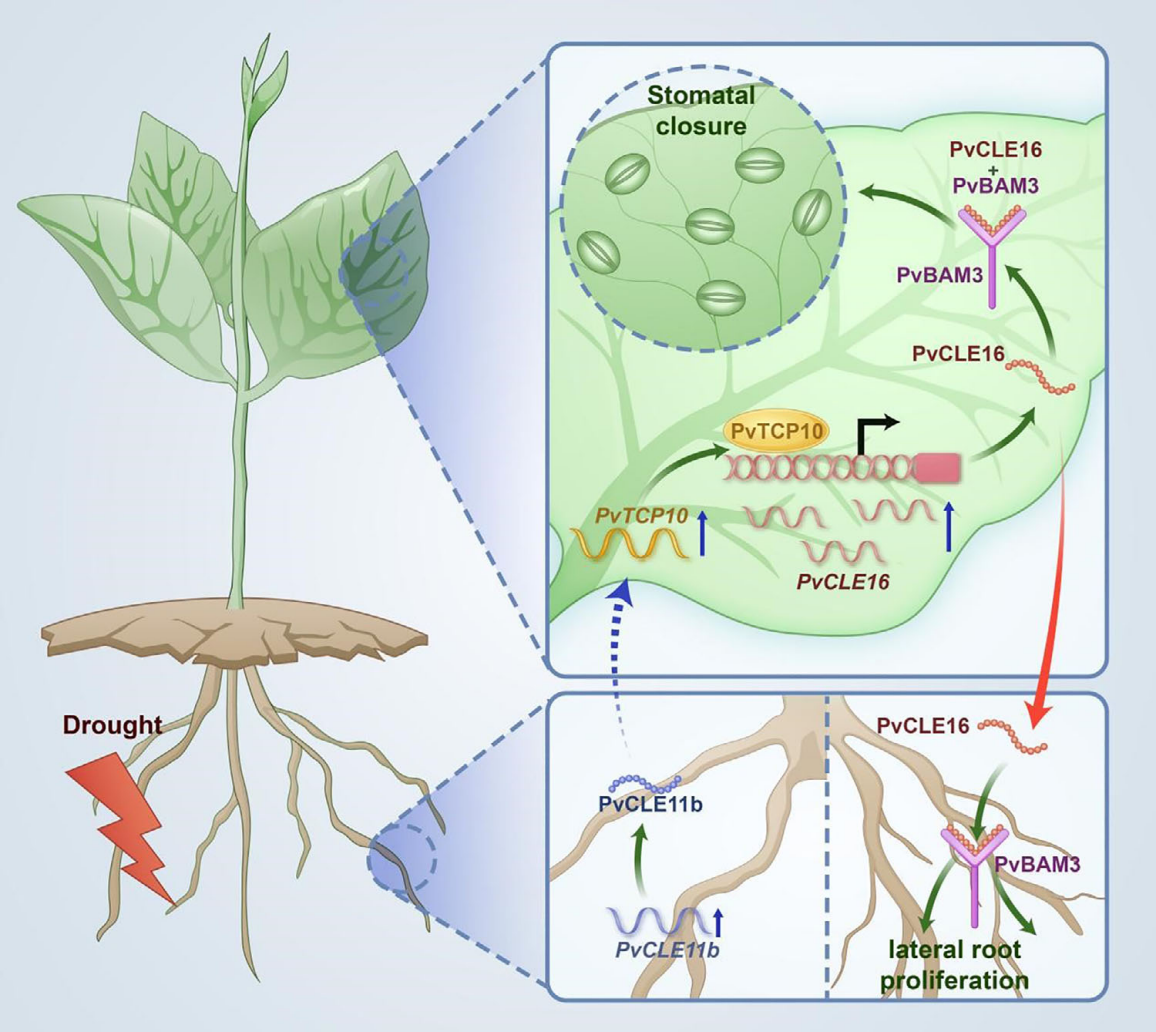
A model illustrating the proposed PvCLE11b‐PvCLE16 signal relay in common bean plants under soil drought. Roots initially perceive drought signals, leading to the upregulation of *PvCLE11b* expression. The encoded peptide then mobilizes from roots to shoots as an acropetal signal, activating *PvTCP10* expression in leaves. This, in turn, transcriptionally upregulates *PvCLE16*. The PvCLE16 peptide, upon perception by its receptor PvBAM3, induces stomatal closure in leaves. When transmitted basipetally (shoot‐to‐root), it suppresses primary root elongation while promoting lateral root proliferation. Together, this PvCLEs‐mediated signaling network orchestrates a coordinated root‐shoot response to soil drought, enhancing the plant's drought adaptation.

In many plant species, including common bean, exposure to moderate soil drought triggers a suite of morphological and physiological adaptations aimed at conserving water, minimizing energy expenditure, and enhancing root‐mediated water uptake [62, 63]. Tightly orchestrated communication between shoots and roots is essential to maintain these functional adaptations. Signaling molecules, including phytohormones, small peptide hormones, calcium waves, inorganic ions, as well as signaling of osmotic changes and electric currents, have been proposed as root‐to‐shoot signals in drought response [15 ^,^ 64, 65, 66]; however, no shoot‐derived signal had previously been identified that feeds back to the roots to coordinate drought responses. In this study, we identified PvCLE16, a member of the CLE family of small peptide hormones, as a shoot‐derived signal specifically induced in leaves and transported rootward under moderate soil drought, filling a critical gap in our understanding of drought signaling networks. Locally in leaves, PvCLE16 contributes to stomatal closure, thereby reducing water loss. Additionally, PvCLE16 acts as a mobile peptide that translocates to the roots, where it inhibits primary root elongation and promotes lateral root development, a phenotype considered adaptive under moderate drought, when shallow soil moisture is still available but limited. Thus, PvCLE16 represents a previously unrecognized shoot‐to‐root signal that coordinates whole‐plant drought adaptation, broadening our understanding of CLE peptide‐mediated systemic responses to environmental stress. Our results also suggest the presence of an upstream root‐derived signal, PvCLE11b, which activates the transcription factor PvTCP10 and its downstream target *PvCLE16* in leaves. Silencing *PvCLE16* in leaves abolished stomatal closure in response to root‐applied synthetic PvCLE11bp. Conversely, silencing *PvCLE11b* in roots impaired drought‐induced *PvCLE16* expression in leaves and its associated shoot responses. Together, these findings support a root‐shoot‐root CLE signaling relay model, in which distinct CLE peptides mediate a hierarchical, bidirectional communication loop (Figure 9).

It is noteworthy that, despite the technical challenges associated with studying mobile signals in common bean, we overcame key limitations through a combinatorial strategy that included mRNA quantification, synthetic peptide application, hairy root transformation, RNA interference, and transient expression. Although repeated efforts to establish a robust and reliable mass spectrometry‐based method for directly quantifying PvCLE16 and PvCLE11b peptides in planta were unsuccessful, due to their low abundance and high sequence similarity to other CLE family members, we observed distinct expression patterns: *PvCLE16* was induced in leaves but not roots under MD conditions, while *PvCLE11b* showed the opposite pattern. Remarkably, each peptide induced significant phenotypes in distant organs, strongly suggesting their inter‐organ mobility. These observations, together with evidence from fluorescently labeled peptide tracing using scrambled peptide controls, support the conclusion that PvCLE16 and PvCLE11b act as bona fide mobile signaling peptides. Moreover, hairy root transformation, which generates composite seedlings with transgenic roots and wild‐type shoots, served as an effective functional alternative to grafting, the gold standard for investigating root‐shoot signaling.

While root‐to‐shoot signaling is widely accepted as the primary route for conveying soil drought stress to aerial tissues, the biological rationale and significance of the shoot‐to‐root signaling is intriguing. Recent studies suggest that such systematic signals fine‐tune complex, environmentally responsive processes [67, 68, 69]. We propose that under natural soil drought conditions, gradual soil drying first induces *PvCLE16* upregulation in leaves, enabling rapid water conservation through reversible stomatal regulation. Accumulating PvCLE16 peptides are then transported to the roots, where they modulate root architecture. This phased response may allow plants to delay energetically costly root morphological adaptations until reaching critical soil drought severity (moderate or more severe), thereby optimizing resource allocation. A fine‐tuning mechanism involving shoot‐to‐root signaling has also been recently revealed in the process of soybean nodulation under UV‐B exposure [67]. This mechanism may bear resemblance to the CLE‐CLV1 pathway regulating root‐knot nematode (RKN) infection. In that process, root‐derived CLEs ascend to the shoot, where they are perceived by CLV1, which in turn induces a secondary shoot‐derived signal that promotes gall formation and RKN development [68, 70]. Such intricate signaling is likely essential for precise, coordinated responses across plant organs under stress. The root‐and leaf‐confined *PvCLE11b* and *PvCLE16* expression, respectively, provide the basis for spatial precision.

Receptors play a key role in CLE peptide signaling. Most CLE receptors are serine/threonine receptor‐like kinases (RLKs) [71]. Through a combination of bioinformatic, biochemical, and transgenic approaches, we identified PvBAM3 as the primary receptor for PvCLE16 mediating drought responses. In *Arabidopsis*, AtBAM3, along with AtBAM1, are known as the receptors of AtCLE25 that regulates dehydration‐induced stomatal closure in leaves [15]. CLE receptors often form homo‐ or heteromeric complexes; for example, CLV1 homodimers bind CLV3, while CLV2‐CRN heterodimers also transduce CLE signals [71, 72]. Thus, PvBAM3 may function as a homomeric complex or potentially as part of a receptor complex that amplifies downstream signaling output. Notably, although our functional analyses support PvBAM3 as a receptor mediating drought responses in both leaves and roots, its overall expression level in whole‐root samples is relatively low. This likely reflects cell type‐specific expression of BAM proteins, as documented in model plants [73]. Future studies, particularly those employing emerging single‐cell omics technologies, will enable a more refined dissection of cell type‐specific ligand‐receptor interactions underlying PvCLE16 signaling. Other RLKs, such as PvTDR, may also interact with PvCLE16, but mediate biological processes other than drought response.

We establish that PvCLE11b functions upstream of PvCLE16, serving as a root‐derived cue that feeds into the PvTCP10‐PvCLE16 signaling module in leaves. However, the mechanisms by which PvCLE11b signaling is perceived and transduced to activate *PvTCP10* expression remain not fully solved. Our preliminary analyses suggest that PvTCP10 exhibits transcriptional self‐activation activity (Figure S12), similar to other TCP‐type TFs [74]. Previous studies further demonstrate that the transcriptional activity of TCP proteins can be modulated by protein phosphorylation [75, 76]. Given that CLE peptides are typically perceived by receptor‐kinase module [73, 77], it is likely that PvCLE11b is recognized by a receptor kinase and activates PvTCP10 through phosphorylation‐dependent mechanisms. Substantial further work will be required to fully elucidate this upstream signaling process.

In conclusion, CLE peptides are evolutionarily ancient and highly conserved across a wide range of plant lineages, from green algae to angiosperms, highlighting their profound functional and evolutionary importance [78]. Our findings demonstrate that systemically secreted CLE peptides mediate the physiological integration of moderate drought stress in common bean, echoing earlier reports in *Arabidopsis* while extending this concept to a crop species. This work adds novel facets to the understanding of CLE peptide signaling and highlights its central role in coordinating environmental cues with developmental and physiological responses. We anticipate that future discoveries will increasingly establish CLE peptide signaling as a central mechanism by which plants integrate environmental cues with developmental and physiological responses. This conceptual advancement will pave the way for leveraging CLE peptides as synthetic tools in crop improvement through signaling pathway engineering.

## 4 Experimental Section

### 4.1 Plant Materials

The common bean (*Phaseolus vulgaris*) cultivar ‘honghuabaijia’ and *Nicotiana benthamiana* were used in this study.

### 4.2 Growth Conditions and Stress Treatments

Common bean seeds were germinated at 25°C, and the resulting seedlings were grown in pots containing a peat‐vermiculite mixture within a greenhouse. The greenhouse conditions were tightly controlled, maintaining a constant temperature of 25°C, 60% relative humidity, and a 14/10 h light/dark cycle, with regular irrigation.

To impose progressive soil drought, tray‐cultivated four‐week‐old common bean seedlings were transferred to the ‘PlantArray’ phenotyping platform (Figure 1A) as described in our previous study [79]. The soil‐plant‐atmosphere parameters including soil water content, Vapor Pressure Deficit (VPD), photosynthetically active radiation and temperature were monitored automatically by the system [80].

For osmotic stress, germinated seeds were transplanted into plug trays filled with perlite, and the seedlings were kept under the same conditions as their soil‐grown counterparts. The 10‐day‐old seedlings were then exposed to a 5% (w/v) PEG‐6000 solution (Biosharp, China) to simulate osmotic stress. *F_v_/F_m_* was measured as previously described [42].

### 4.3 RNA Isolation, RT‐qPCR, and RNA‐Seq

Total RNA was extracted from *Phaseolus vulgaris* tissues (roots and leaves) using TRIzol reagent (Invitrogen, USA). Genomic DNA was removed by DNase I (Takara, Japan) and reverse transcription was performed with 1 µg RNA using PrimeScript RT Master Mix (Takara, Japan) as previously described [81]. RT‐qPCR was performed using the qTOWER 3 Real‐Time PCR detection system (Analytik Jena, Germany) in a 20 µL reaction containing 2 µL of 7‐fold‐diluted cDNA, each primer at 5 µM, and TOROGreen qPCR Master Mix (Toroivd, China). The housekeeping gene *PvUBI* (*Phvul.007G052600*) was employed as the reference for normalization in the delta‐delta cycle threshold method, quantifying relative expression levels. For all results, data represent means ± SD from three independent plants.

RNA‐Seq libraries were constructed from three biological replicates per time point using the NEBNext Ultra II RNA Library Prep Kit (NEB, USA). Sequencing was performed on an Illumina NovaSeq 6000 (150 bp paired‐end). Raw reads were processed with Trimmomatic v0.39 (Q20 filtering), aligned to the *Phaseolus vulgaris* genome (v2.1, Phytozome) via HISAT2 v2.2.1, and quantified using StringTie v2.1.4. Differential expression analysis (DESeq2 v1.30.1) identified genes with |log2FC| >1 and FDR <0.05, with specif gene expression validated against RT‐qPCR data [40].

### 4.4 In Silico Identification of PvCLE Genes

The *PvCLE* genes were identified by searching the *Phaseolus vulgaris* genome on Phytozome (*Phaseolus vulgaris* v2.1, https://phytozome.jgi.doe.gov/) using known CLE precursor peptides from *Arabidopsis thaliana*, with Hidden Markov Models (HMMs) as previously described [82, 83]. A phylogenetic tree was constructed from multiple sequence alignments using MAFFT (v7.471) with default parameters. The tree was generated using the maximum likelihood method, with 1000 bootstrap replicates to assess branch support [84].

### 4.5 Hairy Root Transformation

The full‐length CDSs of *PvCLE16*, *PvBAM3* and *PvTDR* were amplified and integrated into the modified binary vector pMDC83, driven by CaMV 35S promoter, respectively. These vectors were then introduced into *Agrobacterium rhizogenes* strain K599. Hairy roots were induced according to published protocols [85] according to published protocols. Hairy roots exhibiting GFP fluorescence were identified as transgenic roots.

### 4.6 In Planta Transient Overexpression and RNAi

The full‐length CDSs of *PvTCP10*, *PvBAM3*, *PvTDR* were amplified and cloned into the pCV‐GFP vector under the control of the 35S promoter as previously described [86, 87]. Fragments corresponding to the first 1800 bp of the N‐terminal region of GUS were amplified to create the *GUS1800‐GFP* constructs, as the negative control. The PMDC83 vector mentioned above was used for transient overexpression of *PvCLE16*, *EV* is set as negative control. Transient overexpression in *Phaseolus vulgaris* leaves was performed using the *Agrobacterium tumefaciens* strain GV3101 and a vacuum infiltration method, following the protocol outlined by [41].

For RNAi of *PvTCP10*, *PvBAM3, PvCLE11b* and *PvCLE16*, optimal silencing regions were identified using the SGN VIGS Tool (https://vigs.solgenomics.net/). A 300 bp segment of each gene, along with its reverse complement, was amplified and cloned into a modified pFGC‐5941 vector to form a hairpin structure. In the modified vector, the *Bar* gene was replaced with *GFP* as a selectable and fluorescent marker for easy visualization. Transient RNAi in leaves was performed in *Phaseolus vulgaris* leaves following the method described by [41]. The *GUS‐RNAi* construct was used as a negative control. RNAi in hairy root was conducted as mentioned above.

Gene expression of transient overexpression and RNAi were confirmed by RT‐qPCR. At 72 h post‐infiltration (hpi), plants were subjected to a 5% PEG‐6000 treatment, and phenotypic observations were made at 6‐hour intervals following treatment. After imaging, biochemical and physiological analyses were conducted.

### 4.7 CLE Peptide Application, Plant Biochemical and Physiological Phenotyping

Commercially synthesized PvCLE16p and PvCLE11bp were reconstituted in aqueous solution containing 0.05% (v/v) Tween‐20. For foliar treatments, PvCLE16p solutions (0.1, 1, and 10 µM) were uniformly sprayed on both the upper and lower surfaces of common bean seedling leaves following established protocols [88, 89]. Scrambled PvCLE16 peptide (SCR‐PvCLE16p, 10 µM) prepared in the same solution served as the negative control. In parallel experiments, 50 µM FAM‐PvCLE16p was applied, with 50 µM FAM‐SCR‐PvCLE16p used as the corresponding control. For the root application, seedling roots were immersed in PvCLE16p (0.1, 1, or 10 µM) or PvCLE11bp (10 µM), with the corresponding scrambled peptides as controls. For fluorescence assays, 50 µM FAM‐labeled PvCLE16p or PvCLE11bp and their respective FAM‐labeled scrambled peptides were used. FAM signals were observed by using a handheld fluorescent lamp (LUYOR‐3415RG, China).

The visual phenotypes of seedlings were documented using a digital imaging system. For each experimental setup, six plants were examined, and the process was independently repeated three times. Representative photographs were selected to illustrate the observed phenotypes. Stomatal conductance measurements were taken with a portable LI‐600 Porometer/Fluorometer (LI‐COR Biosciences, USA), adhering to the guidelines provided by the manufacturer. The assessment of stomatal aperture followed the methodology outlined by Wu et al. [42]. The *F_v_/F_m_* ratio, which reflects the variable to maximum fluorescence, was determined using an Imaging‐PAM chlorophyll fluorometer (IMAG‐MAXI, Heinz Walz, Germany). Root length and leaf area were documented using a digital imaging system and calculated with Image J software. The root fresh weight over the shoot fresh weight was used to determine the root‐shoot ratio. The leaf expansion rate is calculated by dividing the leaf area applied with PvCLE16p by the leaf area before peptide amplification. For all experiments, statistical analyses were conducted using data from a single leaf per plant, with results presented as mean ± SD.

### 4.8 Y1H Assays

The complete coding sequence (CDS) of *PvTCP10* was cloned into the pB42AD vector as described [42]. An artificial sequence consisting of a triple tandem repeat (base pairs × 3) containing the predicted 13‐bp PvTCP10 binding motifs 1 (5′‐CTG GGA CCC ACC T‐3′) and 2 (5′‐CCG GGA CCC ACC T‐3′) from the *PvCLE16* promoter, along with their adjacent 8‐bp sequences, were inserted into the pLacZi vector. Co‐transformed yeast EGY48 cells, harboring the recombinant pB42AD and pLacZi vectors, were grown on selective medium lacking tryptophan and uracil (SD/−Trp/−Ura). The development of blue coloration on the medium supplemented with 100 mg/µL X‐Gal indicated a protein‐DNA interaction.

### 4.9 EMSA Assays

The full‐length CDS of *PvTCP10* was amplified and inserted into the pET28a vector to express a GST‐tagged fusion protein (Table S4). The His‐PvTCP10 fusion protein was purified using amylose resin (NEB, USA). Two biotinylated oligonucleotide probes, 5’CCA AAT CTC CGG GAC CCA CCT TAT GTG GCG ‐3′and 5’‐ATG TGT ATC TGG GAC CCA CCT TAC GTG ACC‐3′, based on the predicted PvTCP10 binding motifs, were synthesized. Additionally, two biotinylated oligonucleotides, 5′‐CCA AAT CTA AAA AAA AAA AAA TAT GTG GCG ‐3′ and 5’‐ATG TGT ATA AAA AAA AAA AAA TAC GTG ACC ‐3′, containing mutated PvTCP10 binding motifs, were used as controls. The probes were incubated with nuclear extract at room temperature for 30 min. The reaction mixture was then separated on a 0.5×TBE 6% polyacrylamide gel at 60 V for 1 h at 4°C and transferred onto Biodyne B nylon membranes (Pall Corporation, USA). Signal detection was performed using a ChemiDoc XRS imager (Bio‐Rad, USA).

### 4.10 Dual‐LUC Assays

The promoter sequence (−1976 to 0 bp) of *PvCLE16* and (−1929 to 0 bp) *PvTCP10* was amplified and cloned into the pGreenII0800‐LUC vector to serve as a reporter construct. *PvTCP10‐GFP* and *GUS‐GFP* (as a negative control) were used as effector constructs. *Agrobacterium tumefaciens* strain GV3101 (OD_600_ = 1.0) carrying both the effector and reporter constructs was co‐infiltrated into *N. benthamiana* leaves as previously described [90]. Luminescence imaging of the entire leaves was performed using a 5,200 Multi‐imaging system (Tanon, China). The experiment was repeated three times, with three plants per assay, and representative images are reported.

### 4.11 AI‐Based Ligand‐Receptor Prediction

To predict the potential receptors for the PvCLE16p, we first retrieved the protein sequences of all known CLE receptors from *Arabidopsis*. These sequences were then used to perform BLAST searches against the *Phaseolus vulgaris* genome to identify the corresponding receptors for PvCLEs (Table S2). Next, the 3D structure and interaction possibilities of PvCLE16 and its corresponding receptors were predicted in AlphaFold3 software [91], followed by visual analysis of the geometric complementarity, electrostatic, and evolutionary information encoded in the predicted structure using Pymol software. A composite score that combines the confidence of individual structural predictions with the predicted binding affinity were used for validation and ranking the interactions.

### 4.12 BiFC Assays

BiFC assay was conducted as previous described [86, 87]). The full‐length CDS of *PvBAM3, PvBAM1, PvTDR* and *PvCLE16* were PCR‐amplified (Table S3). The resulting fragments were cloned into the pCV‐nYFP and pCV‐cYFP vectors and transformed into *Agrobacterium tumefaciens* strain GV3101. The recombinant bacteria were co‐infiltrated into *Nicotiana benthamiana* leaves at a low concentration (OD_600_ = 0.1) to minimize false positives. At 72 hpi, the leaves were imaged using an ECLIPSE Ti2 inverted confocal microscope (Nikon, Japan), with YFP signals visualized at an excitation wavelength of 514 nm (35% laser intensity) and fluorescence emissions recorded between 530–590 nm (700 gain). The experiment was repeated three times for reliability.

### 4.13 MST Assays

The MST assay was performed as described by Jerabek‐Willemsen et al. [92] with modifications. Receptor proteins fused to GFP were transiently expressed in *Nicotiana benthamiana* leaves, and total proteins were extracted using MST‐compatible extraction buffer (50 mM Tris‐HCl pH 7.5, 150 mM NaCl, 10 mM MgCl_2_, 0.1% Nonidet P‐40, 1 mM PMSF, 1× Protease inhibitor). Crude protein extracts were clarified by centrifugation and subsequently filtered through a 0.22 µm membrane to remove debris and aggregates. The GFP‐tagged receptor‐containing protein extracts were then incubated with a serial dilution of synthetic PvCLE16p at indicated concentrations. After equilibration, the mixtures were loaded into hydrophilic‐treated capillaries and analyzed using the Monolith NT.115 system (NanoTemper Technologies) to determine the equilibrium dissociation constant (Kd).

### 4.14 Quantification and Statistical Analysis

GraphPad Prism 8 was used for statistical analysis. Data are represented as mean ± SE in all graphs. The figure legends provide details of the statistics applied.

## Author Contributions

P.X., S.T., and X.W. designed the research. X.K., Z.W., C.H., C.L., and Z.Z. performed the research. K.N., T.S., M.X., S.W., and S.Y. analyzed the data. P.X., S.T., and X.W. wrote the paper.

## Conflicts of Interest

A Chinese patent related to this research has been issued (Patent No. ZL202111175564.5).

## Supporting information




**Supporting File 1**: advs74290‐sup‐0001‐SuppMat.docx.

**Supporting File 2**: advs74290‐sup‐0002‐TableS2.xlsx.

## Data Availability

The data that support the findings of this study are openly available in NCBI BioProject database at https://www.ncbi.nlm.nih.gov/sra/, reference number No. SRP448770, No. SRP585064 and No. SRP659571.
