# Supplementary material for: A CLE11b‐CLE16 Signaling Relay Mediates Root‐Shoot‐Root Crosstalk for Drought Adaptation in Common Bean
Source: Adv Sci (Weinh). 2026 Feb 8;13(22):e74290. doi: 10.1002/advs.74290 (PMC13088281; doi:10.1002/advs.74290)
Supplement: Supplementary file 1 — Supporting File 1: advs74290‐sup‐0001‐SuppMat.docx. [file ADVS-13-e74290-s001.docx]

Supporting Information

**A CLE11b-CLE16 signaling relay mediates root-shoot-root crosstalk for drought adaptation in common bean**

Xinyang Wu, Shiyuan Tao, Zhuoyi Wang, Ting Sun, Zixin Zhang, Su Yang, Min Xu, Chenyi Huang, Siyi Wang, Xubo Ke, Chenze Lu, Kang Ning, Pei Xu*


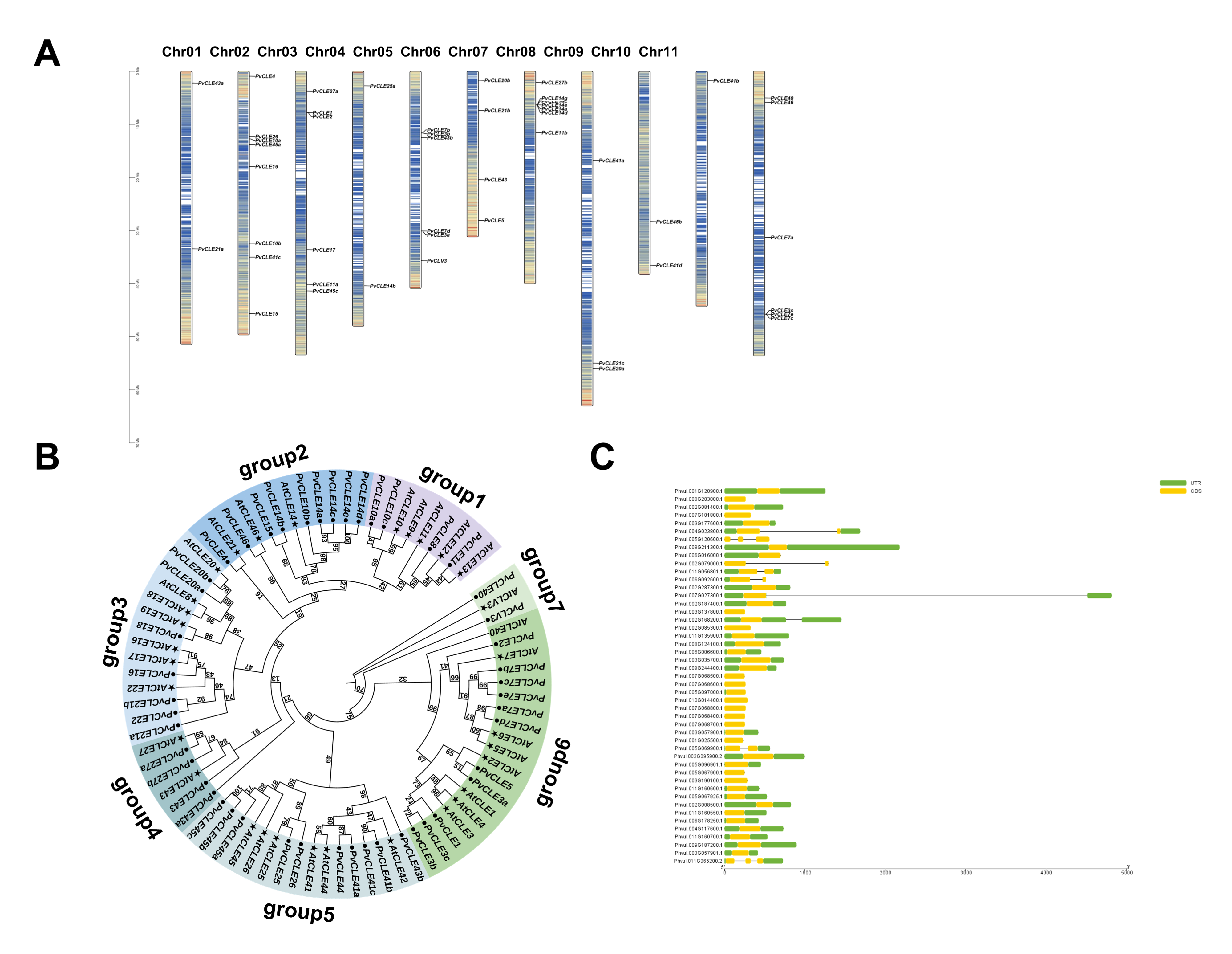


**Figure S1. Genome-wide identification and bioinformatic analysis of the *CLE* family genes in common bean.**

**(A)** Chromosomal distribution of *PvCLE* genes. Chromosome numbers were indicated on the top of each chromosome and Gene ID were shown on the right. The scale on the left was in megabases (Mb).

**(B)** Phylogenetic tree of CLE family members from common bean and *Arabidopsis*. Multiple sequence alignment was conducted with MAFFT (v7.471), and phylogenetic trees were constructed using the maximum likelihood (ML) method in IQ-TREE (v2.0.7). Using iTOL (https://itol.embl.de/) for image beautification.

**(C)** Structural analysis of genes within the *PvCLE* family.


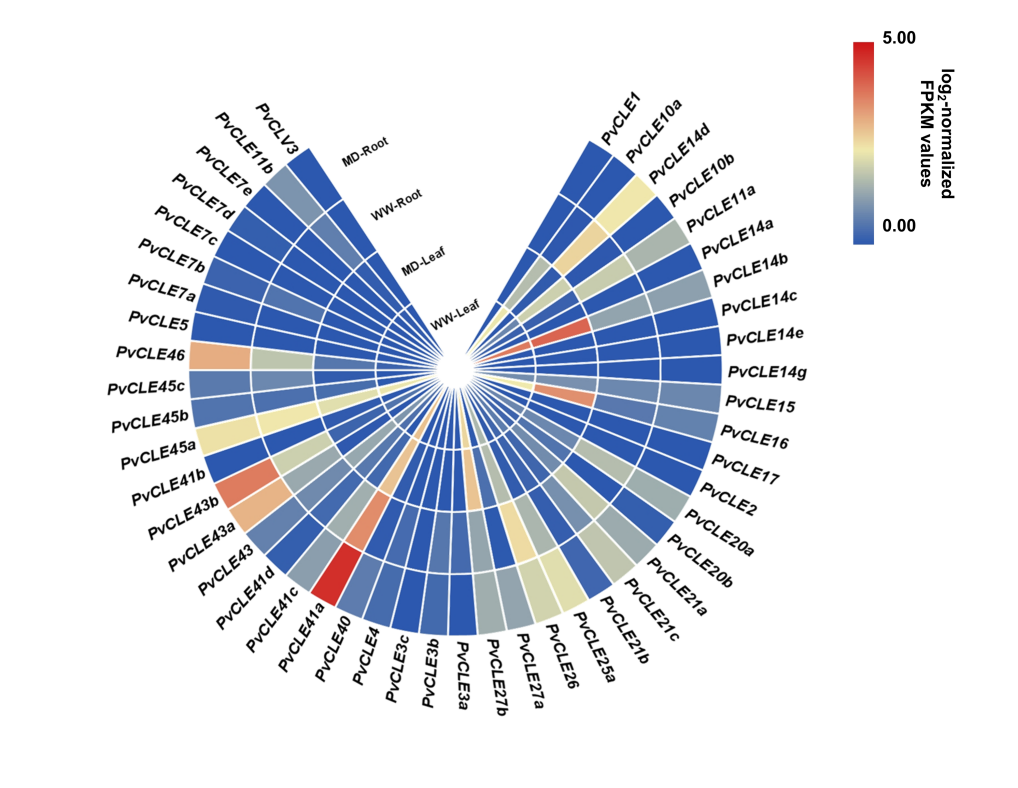


**Figure S2. Heatmap showing the expression profiles of all *PvCLE* genes under well-water (WW) and moderate drought (MD) stress conditions in leaves and roots.**

**
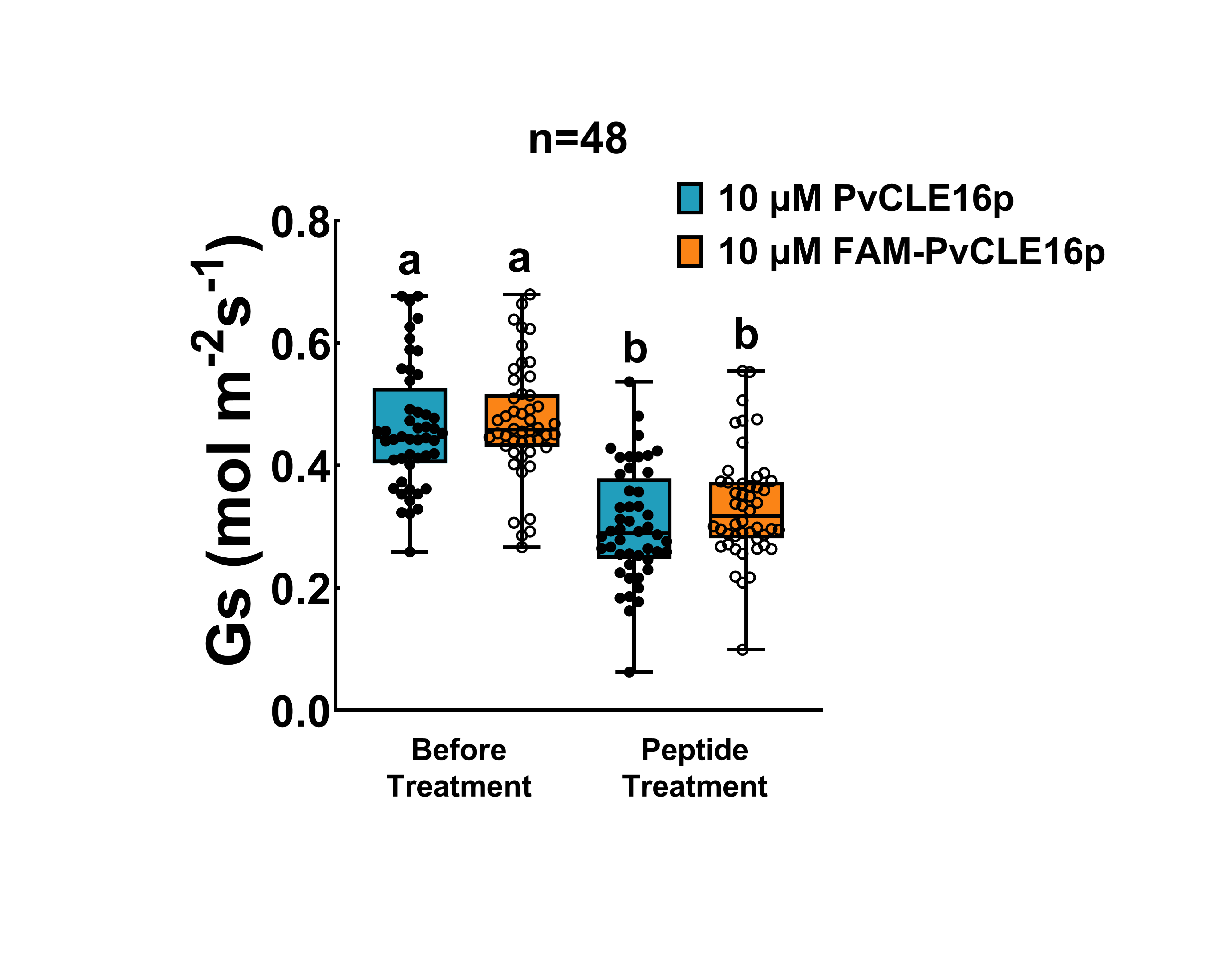
**

**Figure S3. Gs measured before and 2 h after foliar application of PvCLE16p or FAM-PvCLE16p showing that FAM labeling does not affect PvCLE16-induced stomatal closure.** The central line indicates the median, boxes represent the IQR, and whiskers indicate the data range, different letters denote statistically significant differences.


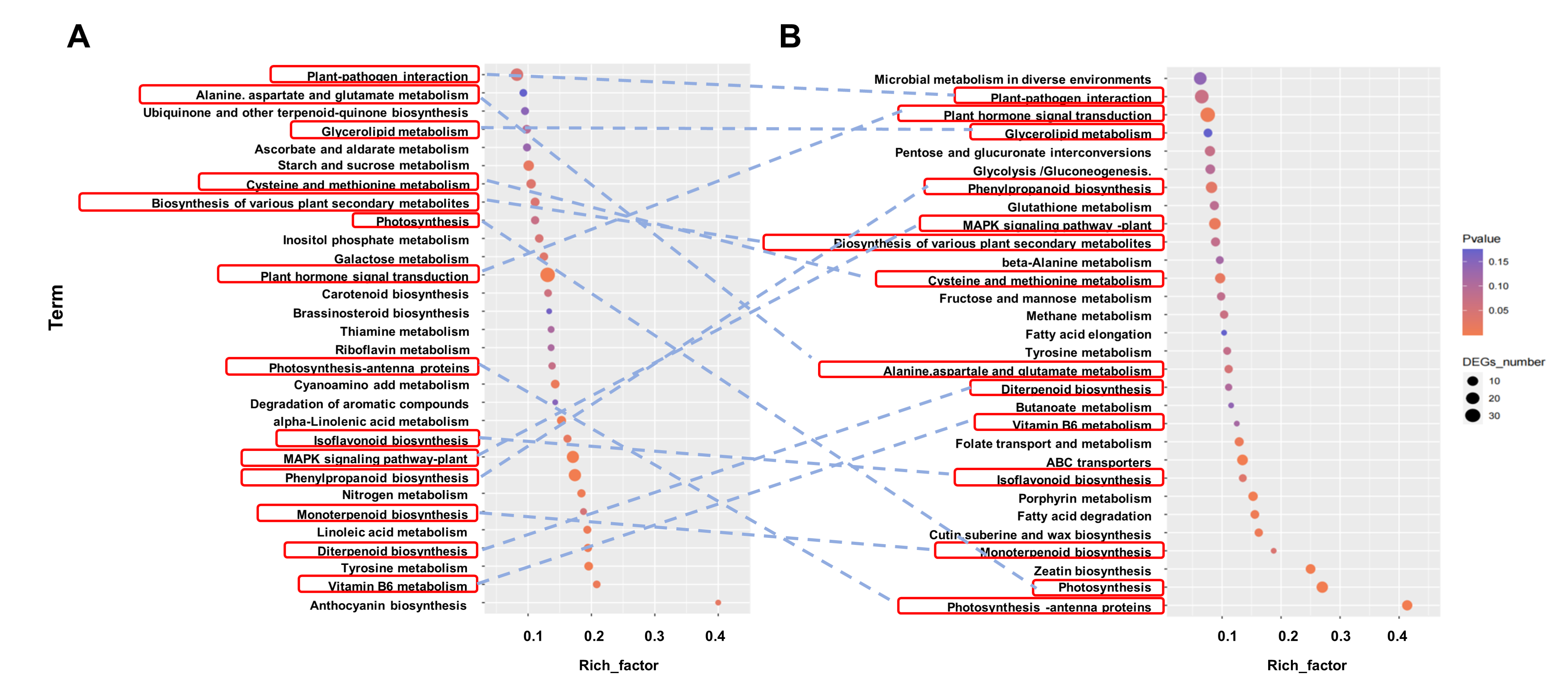


**Figure S4. Comparison of drought-related KEGG pathway enrichments between *PvCLE16*-overexpressing hairy roots and roots of common bean seedlings following leaf application of PvCLE16p.**

1. KEGG pathway enrichment analysis of DEGs in response to *PvCLE16-*overexpeesion in transgenic hairy roots. Bubble size corresponds to the number of DEGs per pathway, and color intensity indicate the enrichment *P* value for the respective pathways.
2. KEGG pathway enrichment analysis of DEGs in roots of common bean seedlings following leaf application of PvCLE16p, compared with SCR-PvCLE16p-treated controls. Bubble size corresponds to the number of DEGs per pathway, and color intensity indicate the enrichment *P* value for the respective pathways.

Functional enrichment analysis of transcriptional responses induced by PvCLE16. Root tissues were harvested for RNA-seq analysis to compare local and systemic transcriptional responses triggered by PvCLE16.


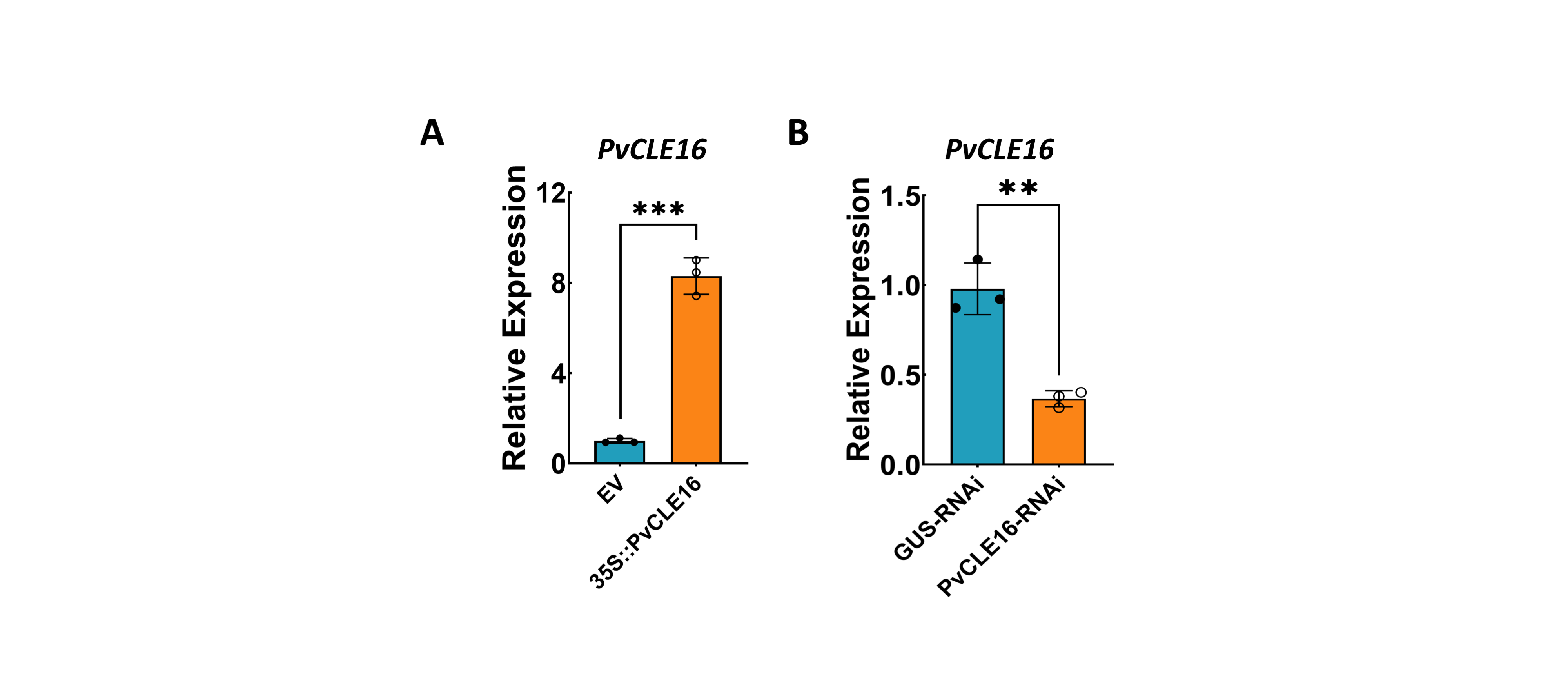


**Figure S5. Expression of *PvCLE16* in *PvCLE16-OE* (A) or *PvCLE16-RNAi* (B) leaves.**

**(A and B)** RT-qPCR analysis of *PvCLE16* expression level in leaves transiently overexpressing *35S::PvCLE16* (A) and expressing *PvCLE16*-*RNAi* constructs (B). Plants expressing *GUS-GFP* or *GUS-RNAi* served as controls. Transcript levels were normalized to *PvUBI* (*Phvul.007G052600*). All data represent mean ± SD. Statistical significance determined by one-way ANOVA with Tukey's test (***P* < 0.01; ****P* < 0.001).

**
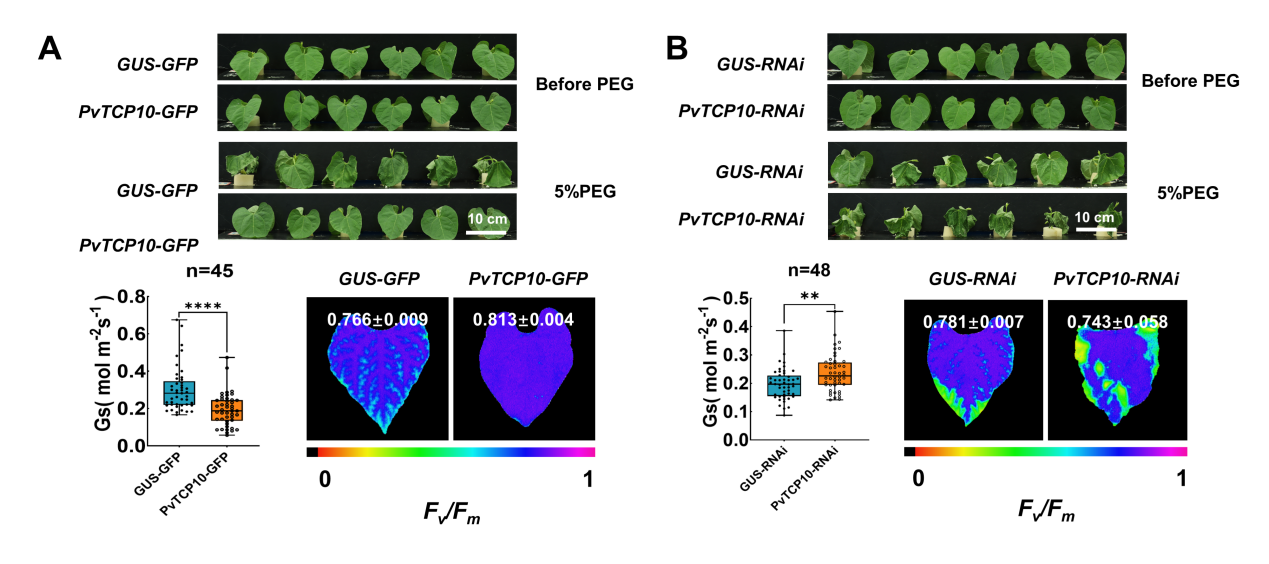
Figure S6. Visual and physiological phenotypes of seedlings overexpressing *PvTCP10-GFP* (A) or *PvTCP10-RNAi* (B) constructs.**

(A) Comparison of visual leaf morphology, Gs and *F_v_/F_m_* in seedlings transiently expressing *PvTCP10-GFP* constructs. Expression of *GUS-GFP* served as the negative control.

(B) Comparison of visual leaf morphology, Gs and *F_v_/F_m_* in seedlings transiently expressing *RNAi-PvTCP10* constructs. Expression of *RNAi-GUS* served as the negative control.

Osmotic stress was imposed by application of 5% PEG (A and B).

In all distribution plots, the central line indicates the median, boxes represent the IQR, and whiskers indicate the data range. Statistical significance was determined by one-way ANOVA followed by Tukey’s multiple comparison test (**P < 0.01; ****P < 0.0001).


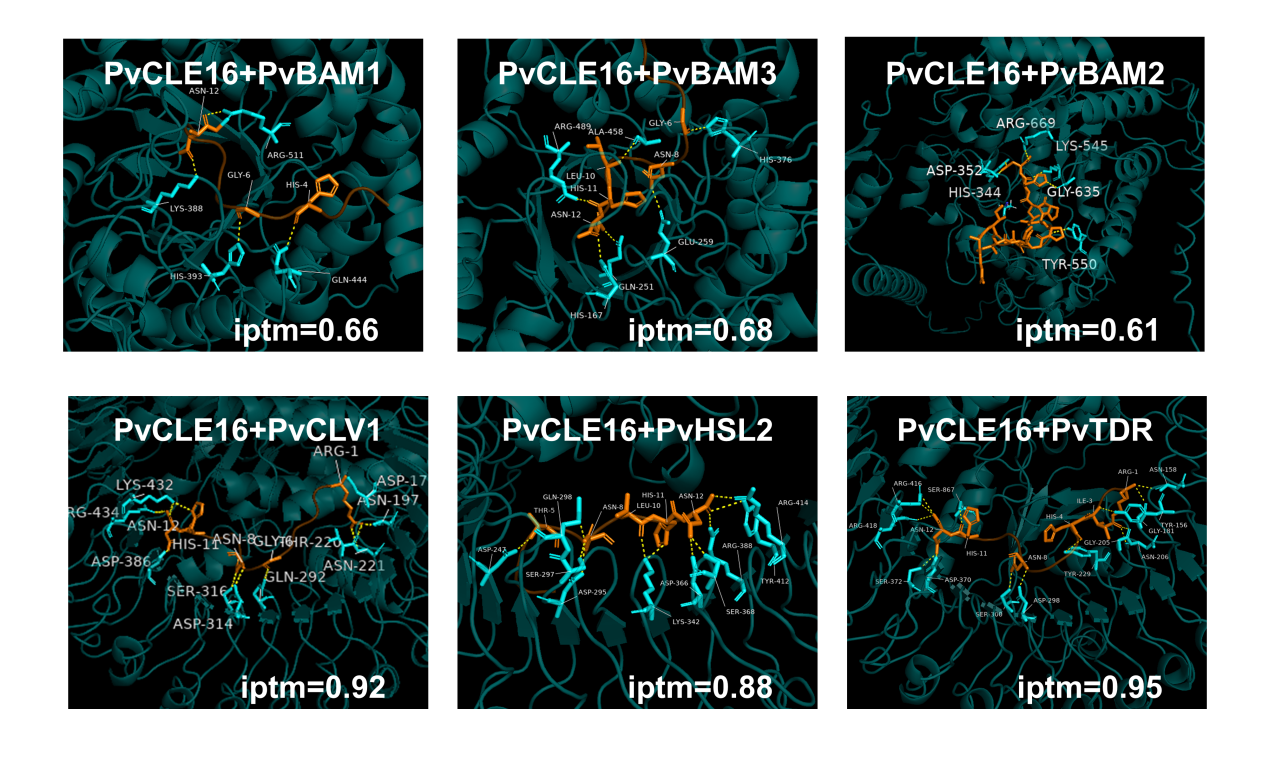


**Figure S7. AlphaFold 3-based prediction of interactions between PvCLE16 and six candidate receptors.**


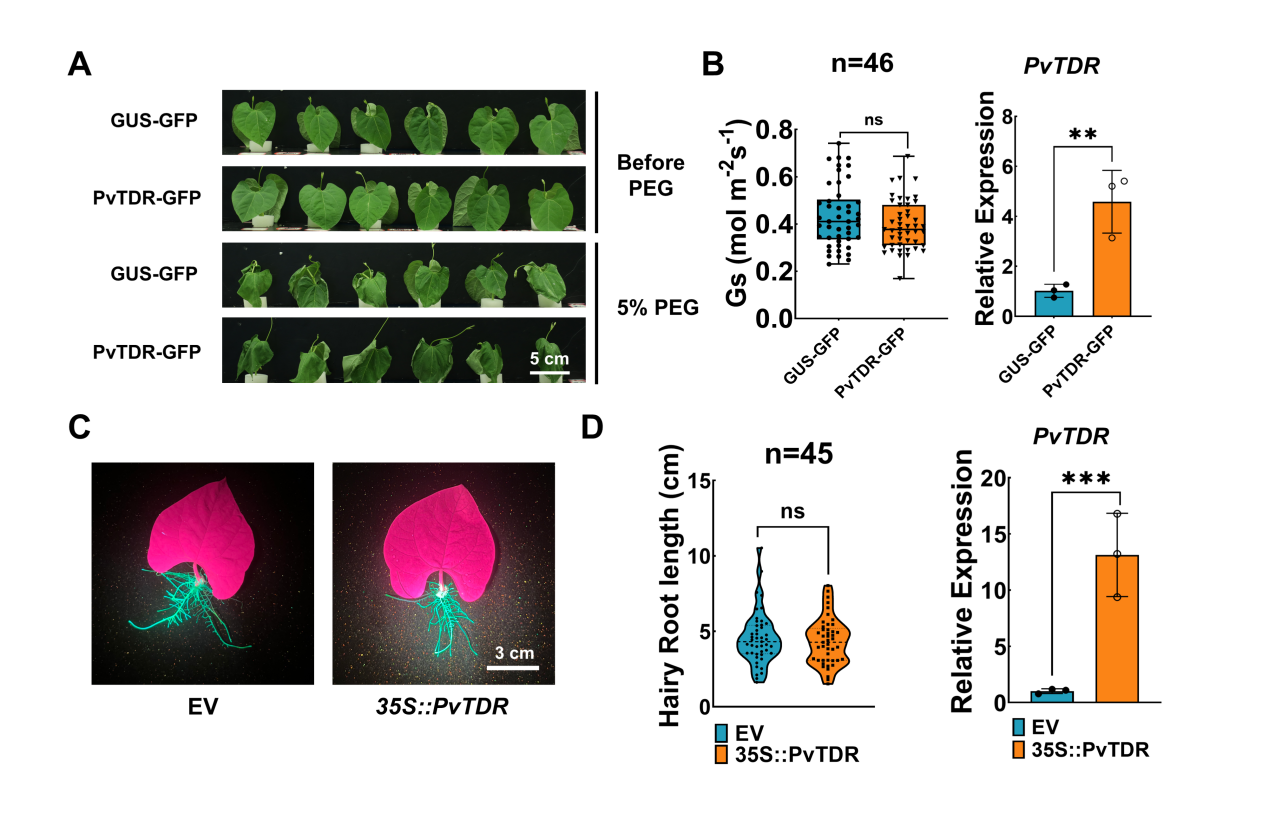


**Figure S8. Functional analysis of PvTDR suggest that it is not required for stomatal or root morphology regulation.**

**(A)** Comparison of visual leaf morphology and Gs in seedlings transiently expressing *PvTDR-GFP* constructs. Expression of *GUS-GFP* served as the negative control. Osmotic stress was imposed by application of 5% PEG.

(**B**) Relative expression of *PvBAM3* in common bean leaves in (A).

(**C**) Morphology and the statistics of transgenic hairy roots length in roots expressing 35S::*PvTDR*, with expressing *EV* as negative control.

(**D**) Relative expression of *PvBAM3* in common bean hairy roots in (C).

In all bar charts, data represent means ± SD.In all distribution plots, the central line indicates the median, boxes represent the IQR, and whiskers indicate the data range. Statistical significance was determined by one-way ANOVA followed by Tukey’s test (***P* ≤ 0.01; ****P* ≤ 0.001; ns: not significant).


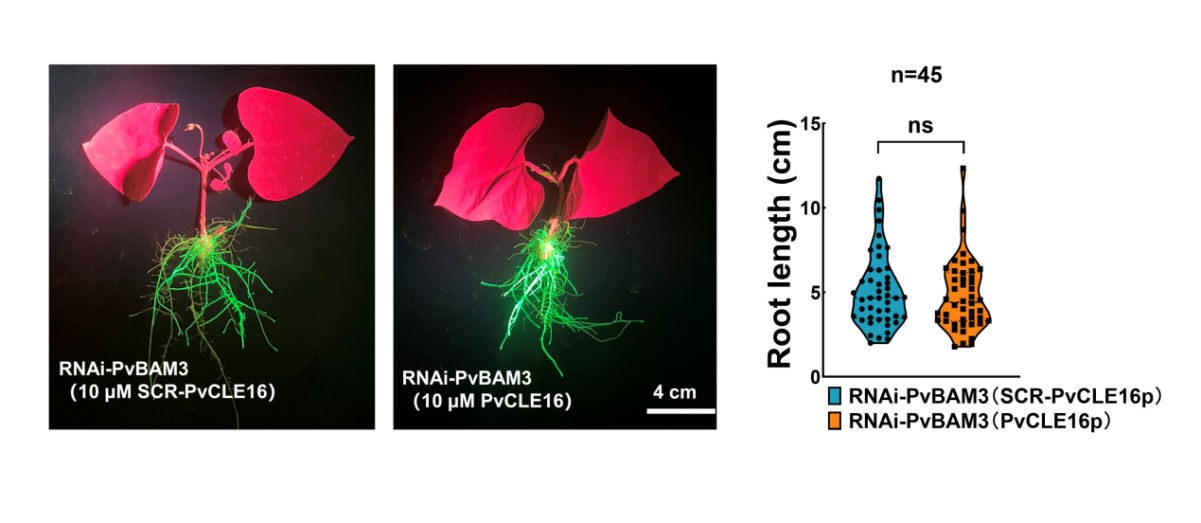


**Figure S9. Root length of *PvBAM3-RNAi* hairy roots after treatment with PvCLE16p or SCR-PvCLE16p.**

The central line indicates the median, boxes represent the IQR, and whiskers indicate the data range. Statistical significance determined by one-way ANOVA with Tukey's test (ns: not significant).

**
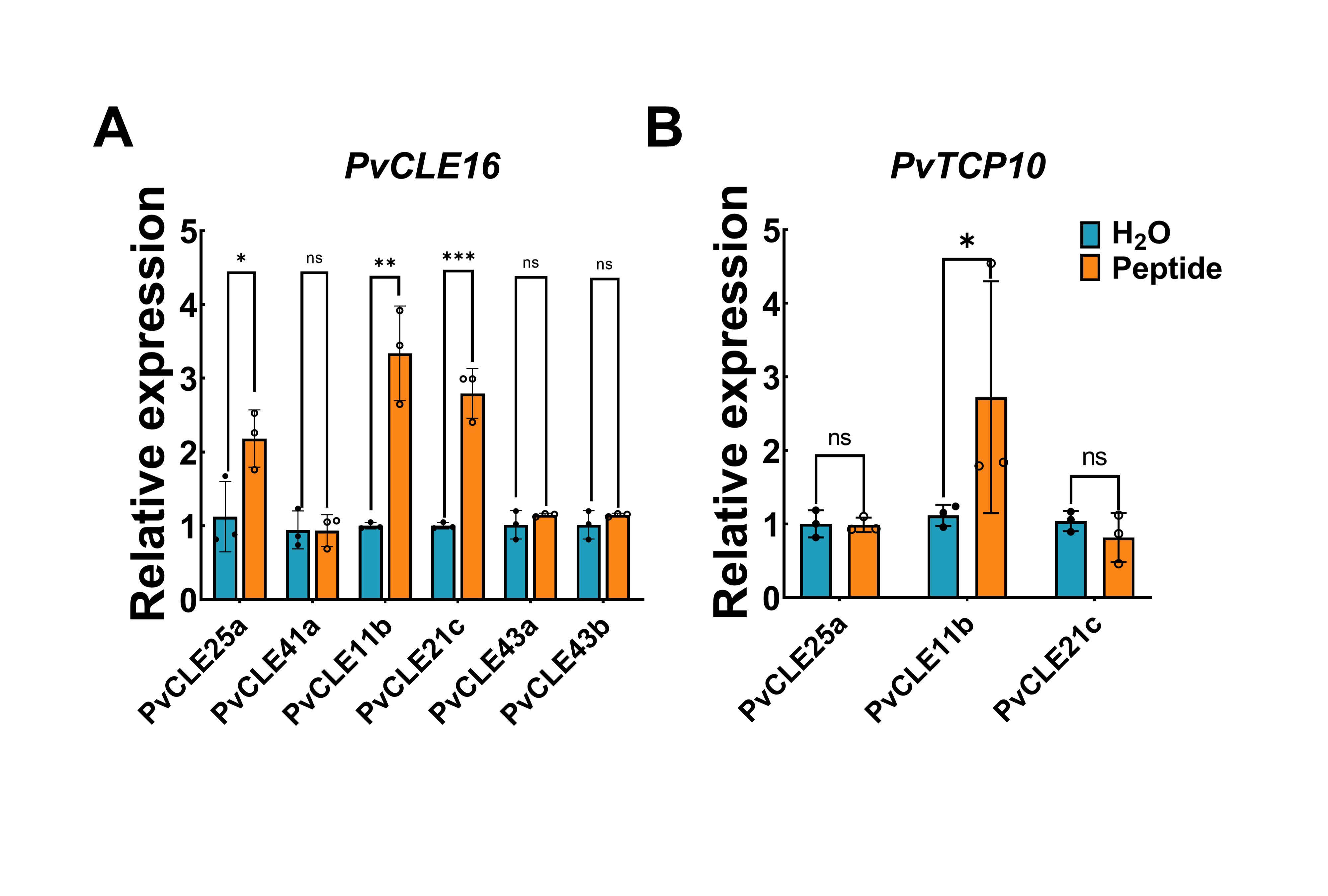
**

**Figure S10. Expression profiles of *PvCLE16* and *PvTCP10* in leaves following root treatment with various PvCLE peptides.**

**(A)** *PvCLE16* expression after root application of individual PvCLE peptides. Three of them - PvCLE25a, PvCLE11b, and PvCLE21c - significantly induced *PvCLE16* expression in leaves.

**(B)** Leaf *PvTCP10* expression in response to root treatment with PvCLE25a, PvCLE11b, and PvCLE21c. All data represent mean ± SD. Statistical significance determined by one-way ANOVA with Tukey's test (**P <* 0.05; ***P <* 0.01; ****P <* 0.001; ns: not significant).


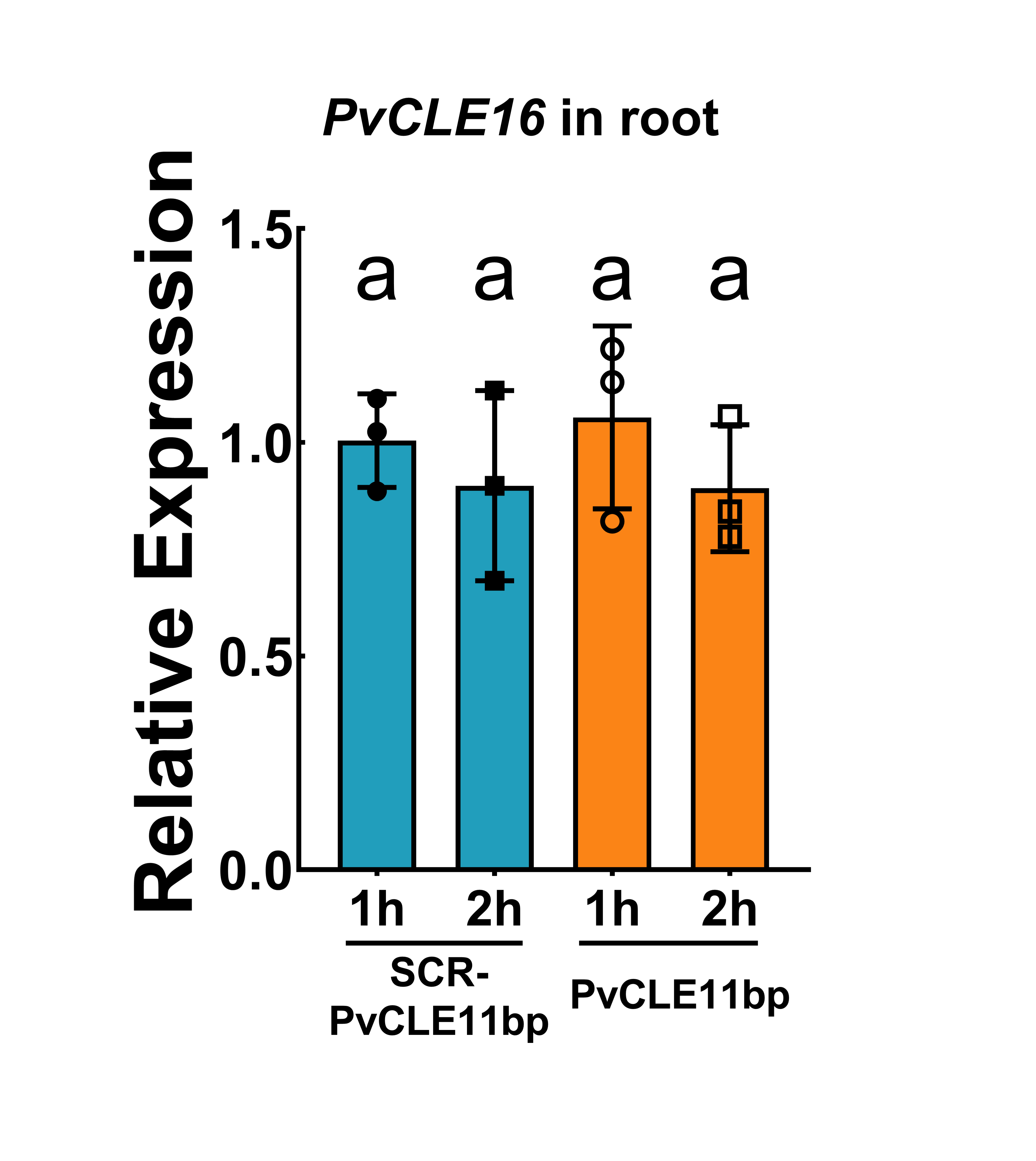


**Figure S11. Expression of *PvCLE16* in root following root treatment with PvCLE11bp (10 μM).** All data represent mean ± SD. Statistical significance determined by one-way ANOVA with Tukey's test with identical letters indicate non-significant differences.


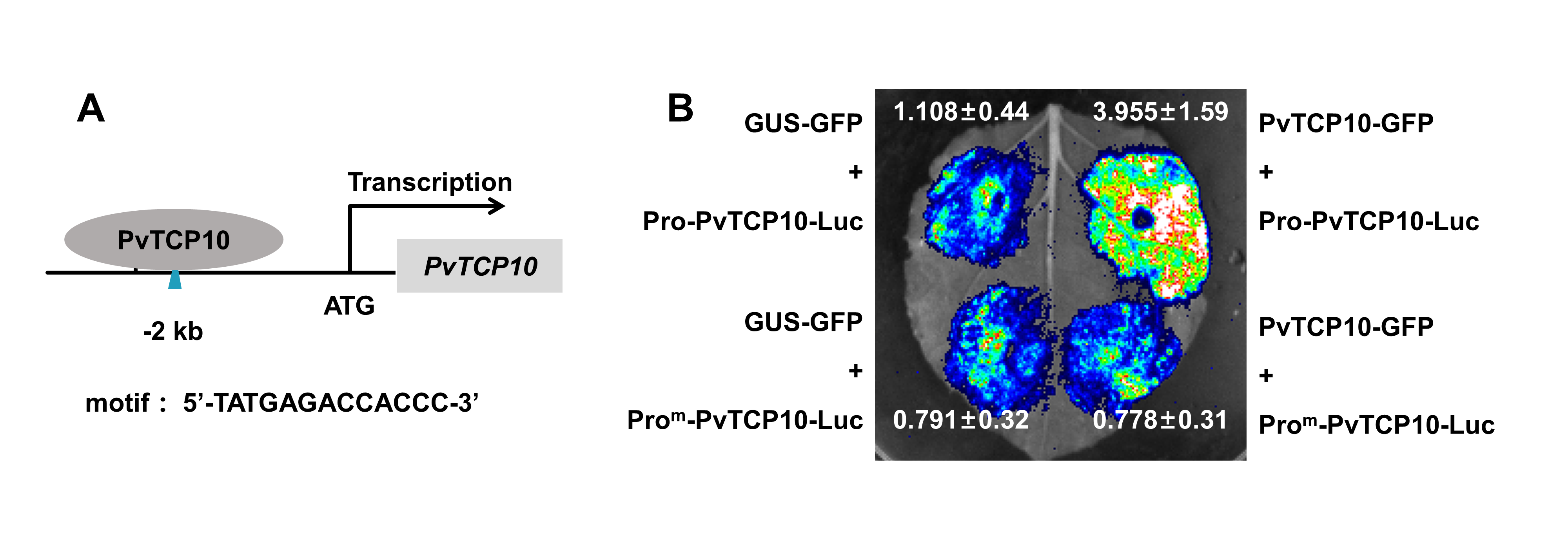


**Figure S12. PvTCP10 exhibits transcriptional self-activation by binding to its own promoter. (A)** Predicted PvTCP10-binding motifs in the promoter region of *PvTCP10*. Numbers indicate positions relative to the transcription start site (+1).

**(B)** Dual-luciferase reporter assay. Motif1 of *Pro-PvTCP10* were mutated. A representative luminescence image of the *N. benthamiana* leaves 60 h after infiltration is shown. The fluorescence intensity was calculated from three independent leaves.

**Table S1. Gene IDs and conserved mature peptide sequences of the PvCLEs.**

| **Name** | **Gene ID** | **Mature peptide sequence** |
| --- | --- | --- |
| PvCLE1 | *Phvul.003G057900* | RLSPSGPDPKHH |
| PvCLE2 | *Phvul.003G057901* | RLSPGGPDAHHH |
| PvCLE3a | *Phvul.005G097000* | RLSPGGPDPKHH |
| PvCLE3b | *Phvul.005G067925* | RLSPGGPDPHHH |
| PvCLE3c | *Phvul.011G160550* | RLSPGGPDPSHH |
| PvCLE4 | *Phvul.002G008500* | RGVPSGANPLHN |
| PvCLE5 | *Phvul.006G178250* | RLAPQGPDPKHH |
| PvCLE7a | *Phvul.011G135900* | RLAPGGPDPQHN |
| PvCLE7b | *Phvul.005G067900* | RLSPAGPDPQHH |
| PvCLE7c | *Phvul.011G160700* | RVSPAGPDPDHH |
| PvCLE7d | *Phvul.005G096901* | RLAPEGPDPHHN |
| PvCLE7e | *Phvul.011G160600* | RVSPAGPDPQHH |
| PvCLE10a | *Phvul.002G081400* | RLVPSGPNPLHN |
| PvCLE10b | *Phvul.002G168200* | RRVPTGPNPLHN |
| PvCLE11a | *Phvul.003G177600* | RLVPTGPNPLHH |
| PvCLE11b | *Phvul.007G101800* | RRVPTGPNPLHH |
| PvCLE14a | *Phvul.007G068600* | RKVPAGPNPLHN |
| PvCLE14b | *Phvul.004G117600* | TATPGGPNPLHN |
| PvCLE14c | *Phvul.007G068400* | VKVPTGPNPLHN |
| PvCLE14d | *Phvul.007G068800* | RLVPSGPNPLHN |
| PvCLE14e | *Phvul.007G068500* | RLVPTGPNPIHN |
| PvCLE14g | *Phvul.007G068700* | KKVPNGPDPIHN |
| PvCLE15 | *Phvul.002G287300* | RDVPGGPNPLHN |
| PvCLE16 | *Phvul.002G095900* | RIIHTGPNPLHN |
| PvCLE17 | *Phvul.003G137800* | RLVPTGPNPLHN |
| PvCLE20a | *Phvul.008G211300* | RRVPTGSNPLHN |
| PvCLE20b | *Phvul.006G006600* | RTVPTGSNPLHN |
| PvCLE21a | *Phvul.001G120900* | RKVYTGPNPLHN |
| PvCLE21b | *Phvul.006G016000* | RKIYTGPNPLHN |
| PvCLE21c | *Phvul.008G203000* | RIIYTGPNPLHN |
| PvCLE25a | *Phvul.004G023800* | RRVPNGPDPIHN |
| PvCLE26 | *Phvul.002G079000* | RRVPNGPDPIHN |
| PvCLE27a | *Phvul.003G035700* | RRVPTCPDPLHN |
| PvCLE27b | *Phvul.007G027300* | RRVPSCPDPLHN |
| PvCLE40 | *Phvul.011G056801* | REVPTGPDPLHH |
| PvCLE41a | *Phvul.008G124100* | HEVPSGPNPISN |
| PvCLE41b | *Phvul.010G014400* | HEVPSGPNPIQN |
| PvCLE41c | *Phvul.002G187400* | HEVPSGPNPISN |
| PvCLE41d | *Phvul.009G244400* | HEVPSGPNPISN |
| PvCLE43 | *Phvul.006G092600* | RVVPSSPDPLHN |
| PvCLE43a | *Phvul.001G025500* | RKVPNASDPLHN |
| PvCLE43b | *Phvul.005G069900* | HEVHSGPNPISN |
| PvCLE45a | *Phvul.002G085300* | RRVPRGSDPIHN |
| PvCLE45b | *Phvul.009G187200* | RTVQKGSDPIHN |
| PvCLE45c | *Phvul.003G190100* | RTVRKGPDPIHN |
| PvCLE46 | *Phvul.011G065200* | RKSPSGPNPVGN |
| PvCLV3 | *Phvul.005G120600* | RKVPSGPDPLHH |

**Table S2. Candidate TFs predicted by PlantTFDB to bind the *PvCLE16* promoter based on motif analysis.**

In a separated excel file.

**Table S3. AlphaFold3-predicted interaction confidence and binding affinities between PvCLE16 and 15 common bean orthologs of *Arabidopsis* CLE receptors.**

| ***P. vulgaris* orthologs to** Arabidopsis **CLE receptors** | **ID** | **ipTM**b | **pTM**a |
| --- | --- | --- | --- |
| PvTDR | *Phvul.011G007200* | 0.95 | 0.64 |
| PvCLV1 | *Phvul.011G042000* | 0.92 | 0.62 |
| PvHSL2 | *Phvul.002G007800* | 0.88 | 0.63 |
| PvBAM3 | *Phvul.003G226900* | 0.68 | 0.78 |
| PvBAM1 | *Phvul.004G143600* | 0.66 | 0.8 |
| PvBAM2 | *Phvul.008G065600* | 0.61 | 0.65 |
| PvHSL1 | *Phvul.002G232600* | 0.52 | 0.63 |
| PvRPK2 | *Phvul.004G037600* | 0.5 | 0.7 |
| PvCLV2 | *Phvul.008G055400* | 0.49 | 0.86 |
| PvPEPR1 | *Phvul.005G184700* | 0.48 | 0.66 |
| PvCRN | *Phvul.008G019200* | 0.42 | 0.71 |
| PvCIK4 | *Phvul.006G174400* | 0.41 | 0.5 |
| PvACR4 | *Phvul.003G038400* | 0.38 | 0.81 |
| PvSERK1 | *Phvul.007G086800* | 0.37 | 0.51 |
| PvBAM9 | *Phvul.006G161200* | 0.34 | 0.76 |

a: predicted template modeling

b: interface predicted template modeling

**Table S4. Primer sequences used in this study.**

| **Gene ID** | **Name** | **Primer name** | **Primer sequence (5'-3')** |
| --- | --- | --- | --- |
| ***Phvul.002G095900*** | ***PvCLE16*** | PvCLE16-CDS-F | ATGATAGGTTTCAGAGAAAGAG |
|  |  | PvCLE16-CDS-R | GTTGTGAAGAGGGTTTGGACC |
|  |  | PvCLE16-RT-F | CCACAACCACATCAAGAACAAGA |
|  |  | PvCLE16-RT-R | GGATCACGCTCAACCACCTT |
|  |  | PvCLE16-Promoter-F | GCCAGTGCCAAGCTTCCAGAACAAAGCTTAAACCAACACAGC |
|  |  | PvCLE16-Promoter-R | TTCTAATTAACTAGTGAGAGGGGAAAAGAGGTAGAGAGTAG |
|  |  | PMDC83-PvCLE16-F | CAGGTCGACTCTAGAGGATCCATGATAGGTTTCAGAGAA |
|  |  | PMDC83-PvCLE16-R | GGGAAATTCGAGCTCGGTACCCTAGTTGTGAAGAGGGTT |
|  |  | RNAi-PvCLE16-BamHI-F | TGGAGAGGACACGCGGGATCCCTCAACCACCTTGGTTTTTTGTT |
|  |  | RNAi-PvCLE16-BamHI-R | GGCGCGCCCCATGCGGGATCCATGATAGGTTTCAGAGAAAGAGAAAGG |
|  |  | RNAi-PvCLE16-XbaI-F | TTGCAGGTATTTGGCTCTAGAATGATAGGTTTCAGAGAAAGAGAAAGG |
|  |  | RNAi-PvCLE16-XbaI-R | GGTCTTAATTAACTCTCTAGACTCAACCACCTTGGTTTTTTGTT |
|  |  | LUC-PvCLE16pro-F | TTCCTGCAGCCCGGGGGATCCCTTCAAAACATTGAACACCACTT |
|  |  | LUC-PvCLE16pro-R | TGTTTTTGGCGTCTTCCATGGATGTAAACAAAAAACATAAAAGTTTGGG |
|  |  | PvCLE16-LUC-mut2-F | ATCTAATTTCAAACAAGTATGTGGCGCAAT |
|  |  | PvCLE16-LUC-mut2-R | ATTGCGCCACATACTTGTTTGAAATTAGATTTGGTGCACAAGTT |
|  |  | PvCLE16-LUC-mut1-R | CACGTACTTGTTTGAAACTATACACATATCAGGGTGT |
|  |  | PvCLE16-LUC-mut1-F | GTGTATAGTTTCAAACAAGTACGTGACCCATCTTTAC |
| ***Phvul.007G052600*** | ***PvUBI*** | PvUBI-RT-F | CAGCTGGAGGATGGAAGG |
|  |  | PvUBI-RT-R | TCCGAACTCTCCACCTCAAGA |
| ***Phvul.004G143600*** | ***PvBAM1*** | PvBAM1-CDS-F | ATGGCGTTGAGCATGACTCA |
|  |  | PvBAM1-CDS-R | GTGCATCAGCACTGCAGCCT |
| ***Phvul.008G065600*** | ***PvBAM2*** | PvBAM2-CDS-F | ATGGCAACTGATATGCAGAGGC |
|  |  | PvBAM2-CDS-R | TACCTGAAGATCAAGAACAGCTTCC |
| ***Phvul.002G007800*** | ***PvHSL2*** | PvHSL2-CDS-F | ATGCCTCAGCCATTGTTTC |
|  |  | PvHSL2-CDS-R | TGGCAGTTTGTGGTCCTTG |
| ***Phvul.011G042000*** | ***PvCLV1*** | PvCLV1-CDS-F | ATGTCAAAAGAAGGAGCAGC |
|  |  | PvCLV1-CDS-R | GAGATTAATGAGGTTGTGGTT |
| ***Phvul.011G007200*** | ***PvTDR*** | PvTDR-CDS-F | ATGAAACCTTTTCTTCTCTTT |
|  |  | PvTDR-CDS-R | GCATTGGTCTGCAACTGG |
| ***Phvul.003G226900*** | ***PvBAM3*** | PvBAM3-CDS-F | ATGGCTCTAACCCTTCGTTC |
|  |  | PvBAM3CDS-R | CACAAGAACAGCCTCTTTT |
|  |  | PMDC83-PvBAM3-F | CAGGTCGACTCTAGAGGATCCATGGCTCTAACCCTTCGT |
|  |  | PMDC83-PvBAM3-R | GGGAAATTCGAGCTCGGTACCTTACACAAGAACAGCCTC |
|  |  | RNAi-PvBAM3-BamHI-F | tggagaggacacgcgggatccTCCCATTGTTACAGTGTCTAGTGGC |
|  |  | RNAi-PvBAM3-BamHI-R | ggcgcgccccatgcgggatccATGGCTCTAACCCTTCGTTCTTC |
|  |  | RNAi-PvBAM3-XbaI-F | ttgcaggtatttggctctagaATGGCTCTAACCCTTCGTTCTTC |
|  |  | RNAi-PvBAM3-XbaI-R | ggtcttaattaactctctagaTCCCATTGTTACAGTGTCTAGTGGC |
| ***Phvul.011G156900*** | ***PvTCP10*** | PvTCP10-CDS-F | ATGGGAGAATCACAGAACCA |
|  |  | PvTCP10-CDS-R | ATGGCGAGAATCAGAGGAAGC |
|  |  | PvTCP10-RT-F | AACAGCAACACCGTCGAGAT |
|  |  | PvTCP10-RT-R | TTTTGCAGTGCAGACCTTGC |
|  |  | RNAi-PvTCP10-BamHI-F | TGGAGAGGACACGCGGGATCCCTCGTCGATGGCCGCCTT |
|  |  | RNAi-PvTCP10-BamHI-R | GGCGCGCCCCATGCGGGATCCTGGGAGAATCACAGAACCACCT |
|  |  | RNAi-PvTCP10-XbaI-F | TTGCAGGTATTTGGCTCTAGATGGGAGAATCACAGAACCACCT |
|  |  | RNAi-PvTCP10-XbaI-R | GGTCTTAATTAACTCTCTAGACTCGTCGATGGCCGCCTT |
|  |  | pJG-PvTCP10-F | GATTATGCCTCTCCCGAATTCATGGGAGAATCACAGAACCACC |
|  |  | pJG-PvTCP10-R | GAAGTCCAAAGCTTCTCGAGTCAATGGCGAGAATCAGAGGAAGC |
|  |  | LUC-PvTCP10pro-F | ttcctgcagcccgggggatccTCTCTGCTGCTATAAACAACCG |
|  |  | LUC-PvTCP10pro-R | tgtttttggcgtcttccatggAATTTTCATTCCCTCTTTC |
|  |  | LUC-PvTCP10pro-mut-F | GGGTGGTCTCATAACAAGTGTCACGATAAGTTC |
|  |  | LUC-PvTCP10pro-mut-R | TATGAGACCACCCATGAATATATAGTGTCATGCAC |
| ***Phvul.007G101800*** | ***PvCLE11b*** | PvCLE11b-CDS-F | CATGTGGCCTTCAAAATCTCTC |
|  |  | PvCLE11b-CDS-R | CATGATGGTGTAATGGGTTTGG |
|  |  | RNAi-PvCLE11b-XbaI-F | ttgcaggtatttggctctagaATGGCCTTCAAAATCTCTCAAGC |
|  |  | RNAi-PvCLE11b-XbaI-R | ggtcttaattaactctctagaCACGCGACGCTTCTCAACC |
|  |  | RNAi-PvCLE11b-BamHI-F | tggagaggacacgcgggatccCACGCGACGCTTCTCAACC |
|  |  | RNAi-PvCLE11b-BamHI-R | ggcgcgccccatgcgggatccATGGCCTTCAAAATCTCTCAAGC |
